# Harmonising dietary datasets for global surveillance: methods and findings from the Global Dietary Database

**DOI:** 10.1017/S1368980024000211

**Published:** 2024-01-19

**Authors:** Dimitra Karageorgou, Laura Lara Castor, Victoria Padula de Quadros, Rita Ferreira de Sousa, Bridget Anna Holmes, Sofia Ioannidou, Dariush Mozaffarian, Renata Micha

**Affiliations:** 1 Friedman School of Nutrition Science and Policy, Tufts University, 150 Harrison Ave, Boston, MA 02111, USA; 2 Food and Agriculture Organization of the United Nations, Rome, Italy; 3 European Food Safety Authority, Parma, Italy

**Keywords:** Dietary data, dietary intakes, harmonisation, 24-h recall, food intakes, nutrient intake

## Abstract

**Objective:**

The Global Dietary Database (GDD) expanded its previous methods to harmonise and publicly disseminate individual-level dietary data from nutrition surveys worldwide.

**Design:**

Analysis of cross-sectional data.

**Setting:**

Global.

**Participants:**

General population.

**Methods:**

Comprehensive methods to streamline the harmonisation of primary, individual-level 24-h recall and food record data worldwide were developed. To standardise the varying food descriptions, FoodEx2 was used, a highly detailed food classification and description system developed and adapted for international use by European Food Safety Authority (EFSA). Standardised processes were developed to: identify eligible surveys; contact data owners; screen surveys for inclusion; harmonise data structure, variable definition and unit and food characterisation; perform data checks and publicly disseminate the harmonised datasets. The GDD joined forces with FAO and EFSA, given the shared goal of harmonising individual-level dietary data worldwide.

**Results:**

Of 1500 dietary surveys identified, 600 met the eligibility criteria, and 156 were prioritised and contacted; fifty-five surveys were included for harmonisation and, ultimately, fifty two were harmonised. The included surveys were primarily nationally representative (59 %); included high- (39 %), upper-middle (21 %), lower-middle (27 %) and low- (13 %) income countries; usually collected multiple recalls/ records (64 %) and largely captured both sexes, all ages and both rural and urban areas. Surveys from low- and lower-middle *v*. high- and upper-middle income countries reported fewer nutrients (median 17 *v*. 30) and rarely included nutrients relevant to diet-related chronic diseases, such as *n*-3 fatty acids and Na.

**Conclusions:**

Diverse 24-h recalls/records can be harmonised to provide highly granular, standardised data, supporting nutrition programming, research and capacity development worldwide.

Malnutrition in all its forms is a leading modifiable risk factor for mortality and morbidity globally^(1–7)^. It is, therefore, essential that scientists, policy makers and other stakeholders are able to characterise the whole diet (food, beverage and nutrient intakes), estimate diet-related health, economic and environmental burdens and inform and implement evidence-based priorities^(2,3,8–12)^. Reliable, comprehensive and regularly collected dietary data in all nations are critical for such work. Given that national patterns hide significant inequalities within countries and populations, it is essential that dietary data are further disaggregated by key subgroups, such as age, sex, ethnicity, socio-economic status and location (e.g. urban or rural)^(13)^. Ideally, dietary data should be nationally representative and collected at the individual level, using validated diet assessment tools, such as 24-h dietary recalls, that collect detailed information on every food item consumed^(14)^. A plethora of national, subnational and community-level nutrition surveys at the individual level have been conducted worldwide^(15)^. However, these are rarely harmonised, hence not comparable between and within countries, populations and over time, due to differences mainly in representativeness, diet assessment tools used and data analysis and reporting.

Over the past 10 years, dietary surveys from around the world have been collated and harmonised at the food group level, e.g. total fruit intake, as part of the Global Dietary Database (GDD). However, such efforts have not previously accounted for the often large variation in the classification and description of individual food items, for example related to differing food definitions, preparation methods, local food names and more. For example, a food may be described by a simple food name (e.g. chicken), but not capture multiple additional levels of detail (e.g. baked or fried; light or dark meat; with or without skin and with or without added oil or salt). Local food names can also denote different foods in varying countries or even within the same country (e.g. biscuit in the USA *v*. the UK). Such heterogeneity can lead to inconsistent food names, definitions, groupings, food matching (referring to the process by which a food is assigned to its corresponding nutrient content using a food composition table or database) and ultimately discrepancies in analysis and reporting. Furthermore, inaccurate or inconsistent food aggregation (or classification) of individual food items with similar characteristics into larger food groups/ categories in a hierarchical manner can compound the errors. This could include, for example, heterogeneity in classifying a food (e.g. avocado and tomato) as a fruit *v*. vegetable or in different levels of food groups (e.g. total vegetables and starchy vegetables). Accurate food classification is especially crucial for mixed dishes and packaged foods, which are an increasing part of the global food supply and must be disaggregated into their ingredients in a standardised fashion to capture intake from all sources. Moreover, even if standardised, such dietary data are rarely publicly available, limiting use by and impact for national and global nutrition communities^(16)^.

To address these critical gaps and advance the quality and quantification of dietary intakes worldwide, the GDD, in collaboration with the European Food Safety Authority (EFSA) and the Food and Agriculture Organization of the United Nations (FAO), developed and implemented comprehensive and standardised methods to streamline the collation, harmonisation and public dissemination of primary individual-level dietary datasets worldwide.

## Methods

### Study overview

The methods for this work build and expand upon the approach of the GDD 2018 to identify, collate and standardise existing dietary data and create a comprehensive and comparable database of diet intakes globally^(15)^. In brief, GDD systematically searched for and identified surveys with quantitative intake data for any of fifty-four foods, beverages and nutrients of interest; extracted intakes using standardised definitions and units per dietary factor, jointly disaggregated by age (23 age groups from 0-6 months to 99+ years), sex, education (low, middle and high), urban/rural residence and pregnancy/lactation status, as available and incorporated these inputs along with relevant covariates into a Bayesian hierarchical prediction model to estimate stratum-specific mean intakes worldwide.

**Fig. 1:**
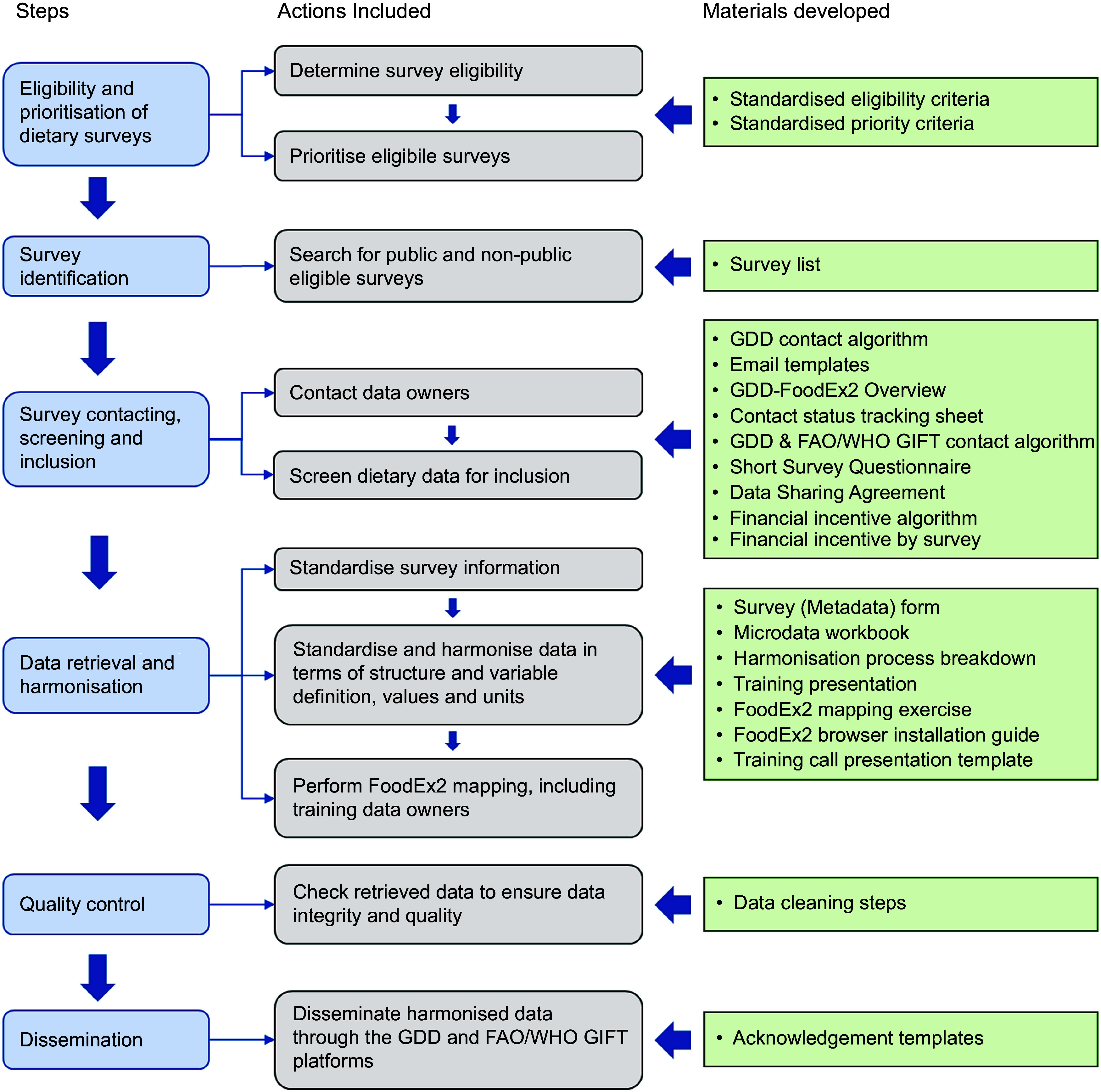
The GDD harmonisation process: steps, actions, and developed material. The Global Dietary Database aimed to harmonise dietary surveys with quantitative 24-h recall or food record data from around the world. For details on material description and use, refer to Table S3

This current project expanded these methods and protocols^(17–19)^ to identify, retrieve, harmonise and disseminate individual-level 24-h dietary recall or record data at their finest level (hereinafter referred to as *microdata*). Standardised criteria, processes and materials – as described in detail below – were developed (Fig. 1, see online supplementary material, Supplemental Table 1).

### Collaborations

Given the shared goal of harmonising individual-level dietary data worldwide, the GDD joined forces with FAO and EFSA^(20)^. In 2017, FAO and the WHO developed the FAO/WHO Global Individual Food consumption data Tool (FAO/WHO GIFT)^(16,21)^, aimed at harmonising and disseminating individual-level dietary microdata, as well as presenting summary statistics in the areas of food consumption, nutrition and food safety^(16)^. EFSA, a European agency providing independent scientific advice on food-related risks, developed and maintains the FoodEx2 food classification and description system (FCDS) and has been working, for over 10 years, on the harmonisation of dietary microdata. Each initiative developed and used their own processes and materials with regards to data harmonisation and dissemination. Yet, through our collaboration, we aimed to maximise efficiency by exchanging resources, methodological approaches and complementary experience and expertise.

### Eligibility and prioritisation of dietary surveys

Surveys were eligible if they had valid quantitative 24-h recall or food record data and aiming to assess the general population of any country (see online supplementary material, Supplemental Table 2). Surveys with less than 100 participants, evidence of strong selection bias (e.g. special population subsets such as with medical conditions), published before 1980, or not agreeing to make the harmonised microdata publicly available through the GDD platform were excluded. Surveys needed to include a minimum level of detail required for *FoodEx2 mapping* (i.e. use of the FoodEx2 FCDS to code all reported foods): specifically, foods should not be reported as ‘generic’ terms (e.g. fruit instead of apple, orange, etc.; legumes instead of beans, lentils, etc.).

Due to the time- and resource-intensive nature of FoodEx2 mapping (see below), the GDD aimed to ultimately include and harmonise 40–50 dietary datasets. Surveys were prioritised based on key characteristics, such as being nationally representative and from populous countries (see online supplementary material, Supplemental Table 3) and aiming to include low (LIC), lower-middle (LMIC), upper-middle (UMIC) and high (HIC) income countries^(22)^. Sub-national and community-level surveys were considered if national data were unavailable for a priority country or if the FoodEx2 mapping had been already performed. Surveys that were more recent, included both men and women and diverse ages, and captured both rural and urban areas were further prioritised. For publicly available surveys, those with datasets in English or Spanish that could be readily managed by the GDD investigator team were prioritised.

### Survey identification

The GDD searched for public and non-public dietary surveys in multiple electronic databases and through extensive direct communications with data owners, nutrition experts and collaborators worldwide. The survey lists of the GDD 2018^(15,23)^, EFSA^(20,24)^ and FAO/WHO GIFT database were screened^(21)^. The GDD 2018 incorporated 1220 public and non-public surveys, including 286 with 24-h recall data covering all world regions. The EFSA database comprised sixty-nine surveys with 24-h recall/ food record data from European countries (fourteen in common with GDD 2017) for which harmonisation and FoodEx2 mapping had been completed, but the datasets were not publicly available^(20)^. The FAO had identified 336 surveys with 24-h recall data (110 in common with GDD 2018) for harmonisation^(21)^. Complementary online searches were performed in PubMed and Google to identify more recent surveys through December 2019. The main search terms included: ‘nutrition survey’ OR ‘diet survey’ OR ‘dietary survey’ OR ‘nutrient survey’ AND ’24-h recall’ OR ‘diet recall’ OR ‘food record’ OR ‘diet record’ OR ‘food diary’ AND ‘national’ OR ‘nationally representative’ AND [‘country of interest’].

### Survey contacting, screening and inclusion

Standardised algorithms and email templates (see online supplementary material, Supplemental Table 1, Supplemental Figure 1) were developed for contacting the data owners of eligible surveys. To augment internal and external consistency and data validity, the GDD, in collaboration with FAO, aimed to establish contact with all prioritised surveys, including publicly available ones. Data owners were invited to participate, harmonise and share their data under one of three options: (1) structure their data per the GDD requirements (see next section for details) and perform the FoodEx2 mapping; (2) structure their data for the FoodEx2 mapping to be performed by the GDD investigators or (3) share their original raw microdata to be structured and mapped to FoodEx2 by the GDD investigators.

Interested data owners completed a nine-question Short Survey Questionnaire (see online supplementary material, Supplemental File 1) to confirm 24-h recall or food record data availability and structure. Questions assessed mixed dish disaggregation, English translation, food matching, FoodEx2 mapping, number of unique food items and mixed dishes and data sharing option (of the three described above). One investigator screened responses, with questions resolved by consensus. The Short Survey Questionnaire did not apply to already harmonised EFSA and FAO/WHO GIFT surveys. For eligible non-public datasets surveys, data owning institutions signed a data sharing agreement; for public datasets, eligibility for re-posting was determined by their terms of use.

Data owners were offered remuneration for harmonisation efforts of up to $7700 per harmonised survey; 57 % of data owners accepted remuneration (mean $2584 per survey). The survey-specific amount was determined based on an algorithm, which was informed by the Short Survey Questionnaire and accounted for the complexity of the required efforts.

### Data retrieval and harmonisation

Standardised protocols and forms were developed to retrieve survey information and data in a systematic manner and achieve high-quality harmonisation. Survey information was retrieved using a metadata form – adapted from the FAO relevant one – and it captured, among other characteristics, survey name, country, year(s), sampling methods, representativeness, sample (size, age and sex), diet assessment method (seasons and days covered, single *v*. multiple, portion size estimation aids) and food composition table/database (FCT/FCDB) (see online supplementary material, Supplemental File 2). Individual-level participant and dietary data (termed microdata) were retrieved using a standardised codebook and extraction template, building on those developed by EFSA. The codebook included instructions on how data owners should structure their data; the variables (name, definition and values) to use for reporting participant data, such as socio-demographics (age, sex and education), anthropometry (weight, height and BMI), pregnancy/lactation status, breast-feeding status and physical activity (see online supplementary material, Supplemental Table 4) and the variables (name, definition, unit of measurement and values) to use for the dietary data, which included all food items consumed per participant, meal type (e.g. breakfast, lunch), time and place of consumption, amount consumed and contents of energy and thirty-nine nutrients (see online supplementary material, Supplemental Table 5).

#### Microdata harmonisation

Microdata harmonisation included standardising variable definitions and coding, data structure and food description. For each step, a standardised process (see online supplementary material, Supplemental Figure 2) and instructions (see online supplementary material, Supplemental Figure 3) were developed to maximise validity and consistency. Each variable in the participant and dietary datasets was characterised according to the standardised codebook previously mentioned (see online supplementary material, Supplemental Tables 4 and 5). The dietary data structure included individual food items consumed per participant, each presented in a separate row along with the corresponding amount and nutrient content. Mixed dishes were disaggregated, as possible, into all ingredients and corresponding amounts (e.g. lentils (200 g) = lentils (130 g), tomatoes (50g), onion (10g), olive oil (10g)); if disaggregation was not part of the data collection, data owners were asked to perform it *post hoc*, based on commonly used recipes among the reference population. Nutrients were assigned to each food item and disaggregated ingredients; food matching was performed by the data owners using the FCT/FCDB they considered as more appropriate for their needs. All reported data were retrieved in English. For datasets harmonised by EFSA and FAO, variables were renamed, recoded and retrieved (e.g. nutrient intakes for EFSA datasets and additional socio-demographics, such as education, for all datasets), as appropriate, so that all harmonised datasets included the GDD standardised set of variables.

#### FoodEx2 mapping

Each food item and disaggregated ingredient was coded according to FoodEx2^(25)^, an FCDS including >4540-terms organised across twenty-one top-level food groups, each further split into up to seven levels of food groups or foods (see online supplementary material, Supplemental Table 6) and a twenty-eight-facet description system (see online supplementary material, Supplemental Table 7). FoodEx2 facets include, for example, cooking and other processes (frying, mincing, sugar-coating, etc.), sweetening agent (including low- or non-energetic sweeteners), production method (organic farming, aquaculture, etc.); yet, not all facets are relevant to food consumption (e.g. packaging material is relevant to food safety). All FoodEx2 foods and facet descriptors have a unique five-character alphanumeric code. For all datasets, each individual food item, after mixed dish disaggregation, was mapped to the appropriate FoodEx2 food term. For food items that were further described, as many facets possible were used to describe all additional characteristics reported (see example below).
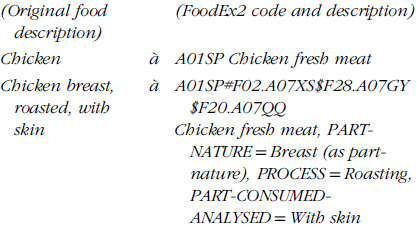



Mapping was performed manually for each food item by a trained coder using the FoodEx2 Catalogue browser^(26,27)^, a desktop application which includes all FoodEx2 foods and facets, allows iterative building of coding and accounts for multiple mapping rules. The GDD investigators received a rigorous 3-day in-person formal training at EFSA’s headquarters^(28)^ and observed a data owner training session delivered by FAO. To enable data owners to also perform high-quality FoodEx2 mapping themselves, a structured training process and relevant materials (see online supplementary material, Supplemental Table 1) were developed; all were reviewed and approved by EFSA. The training materials included a presentation on FoodEx2 theory, concepts, rules and mapping examples (see online supplementary material, Supplemental Figure 3), and a thirty-two-food item exercise to practice the theory learned; EFSA’s FoodEx2 technical report^(25)^ and webinars^(29)^ were further shared as reference materials. Moreover, a 2-hour web call was held to provide clarifications on FoodEx2 mapping and map survey-specific foods. Success of training was validated by asking data owners to map 100 food items from their dataset and hand-checking the results (see online supplementary material, Supplemental Figure 2), with follow-up personalised training as needed.

### Quality control

Data integrity and quality were assessed throughout the retrieval and harmonisation process. All metadata forms were reviewed by one investigator to confirm all fields were completed correctly; with random double checks by a second investigator. Microdata retrieval and harmonisation included specific actions and milestones (see online supplementary material, Supplemental Figure 2), which served as intermediate check points. This included whether datasets had all variables as dictated by the codebook, whether mixed dishes were fully disaggregated, assessment for outliers and errors in amounts consumed based on FoodEx2-code specific EFSA plausibility thresholds and visual inspection of nutrient intakes at the food item level. In addition, all FoodEx2 mapping performed by data owners was reviewed by one investigator for errors with random second reviews of 20–25 % of the foods by a second investigator. For FoodEx2 mapping performed by GDD, up to 50 % of the foods were reviewed randomly by a second investigator. Surveys already harmonised by EFSA and FAO were checked using each initiative’s internal protocols. Data checks were performed using Stata v14·0 (StataCorp LLC). Identified potential errors, inconsistencies or implausible values were discussed within the GDD team, followed by direct communication with data owners to obtain clarifications and additional consultation with EFSA for remaining uncertainties.

### Dissemination

For each survey, the metadata, microdata and corresponding standardised codebook were prepared. All harmonised surveys are publicly available and free to download through the GDD website,^(23)^ with parallel availability through the FAO/WHO GIFT website for consenting surveys; any future harmonised surveys will be added on a rolling basis. Three files are available for each harmonised survey: the participant dataset, the diet dataset and the codebook, which includes the citation of the harmonised survey, survey metadata, all data variables and instructions on how to use FoodEx2 to classify foods into food groups.

## Results

### Survey identification, screening and inclusion

**Fig. 2:**
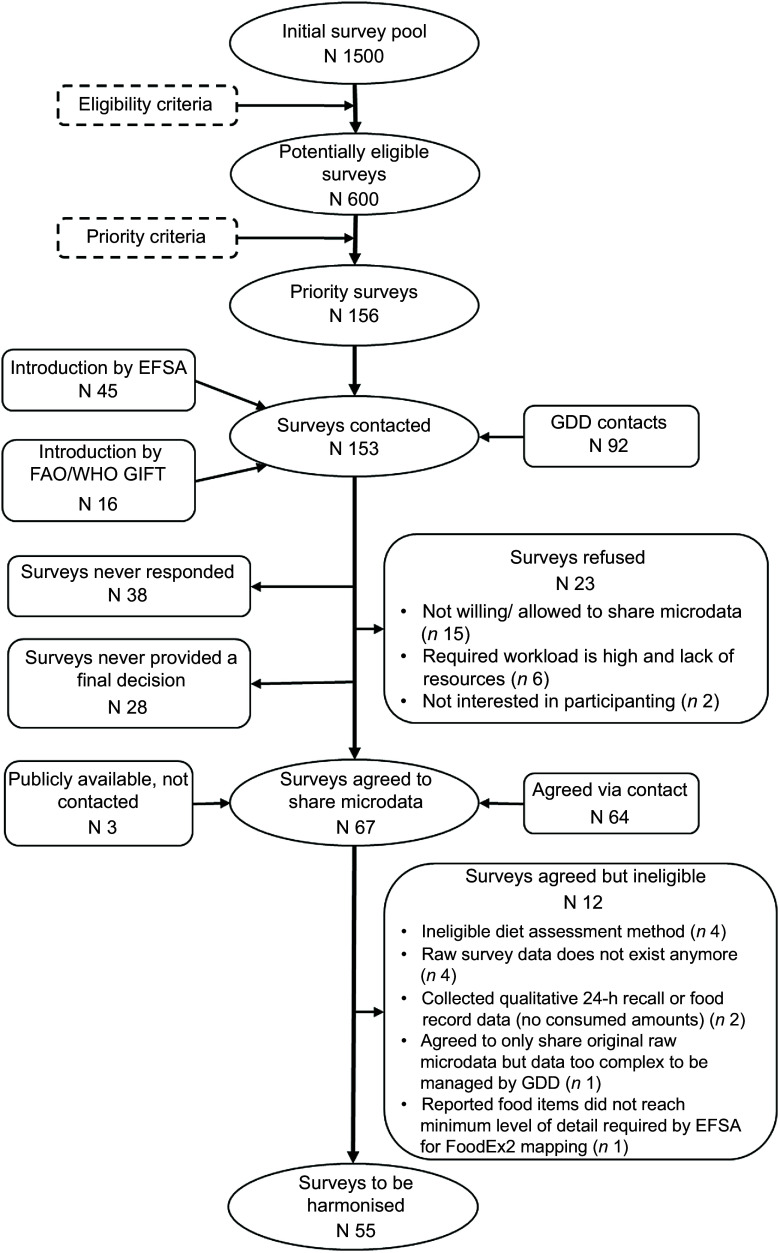
Screening and selection process of dietary surveys with valid 24-h recall or food record data for GDD harmonisation. All public and non-public priority surveys were contacted, with the exception of the three NHANES surveys, for which the harmonisation could be performed independently. Of the 55 surveys included for harmonisation, the harmonisation has not been completed for three of these due to time and resource restrictions

Of 1500 surveys identified, 600 were eligible for inclusion (Fig. 2). Of those, GDD prioritised and contacted 156 surveys. Of the 156 surveys, twelve (8 %) were excluded based on the Short Survey Questionnaire, e.g. related to qualitative data collection or microdata no longer available; twenty three (15 %) refused to participate; thirty eight (25 %) never responded and twenty-eight surveys (18 %) responded but never made a final decision about participating or not. Overall, fifty-five surveys (35 %) were ultimately included for harmonisation, of which forty two (76 %) were not publicly available (Table 1). Of the fifty-five surveys, twenty four were retrieved directly from data owners, fifteen from EFSA and sixteen from FAO/WHO GIFT.

**Table 1:** Characteristics and progress status of the surveys included for harmonisation

Country	Survey name	Year(s)	Representativeness*	Rolling program†	Sample size‡	Age range (years) ‡	Sex‡	Coverage	Country income§	Diet assessment||	Language	Incentive accepted¶	Leading initiative**	Option*†	Status*‡
ARG	Primer estudio sobre el estado nutricional y los hábitos alimentarios de la población adulta de Rosario^(31)^	2012–2013	Community level	No	1200	18–71	M & F	Urban	UMIC	Single 24hR	Spanish	NA	FAO	NA	Completed
BFA	Food Consumption and Iron Status Survey in the Rural Sourou and Sanguie Provinces^(32)^	2010	Sub-national	No	960	2·6–6 19–55	M & F F	Rural	LIC	Multiple (x2) 24hR	French or local languages	NA	FAO	NA	Completed
BGD	HarvestPlus Bangladesh Bio-fortified Rice Project^(33)^	2007–2008	Community level	No	475	2–70	M & F	Rural	LMIC	12hR & 12h observation/weighing	Bengali	NA	FAO	NA	Completed
BGD	Bangladesh Integrated Household Survey (BIHS)^(34)^	2011–2012	National	Yes	22 173	0–120	M & F	Rural	LMIC	Single 24hR	Bengali*||	NA	GDD	C	Completed
BGR	National Survey of Food Intake and Nutritional Status (NSFIN)^(35)^	2004	National	Yes	2282	16–95	M & F	Urban & Rural	UMIC	Single 24hR	Bulgarian	Yes	EFSA & GDD*§	NA	Completed
BGR	Nutrition of Children Survey (NUTRICHILD)^(36)^	2007	National	No	1723	0–4·9	M & F	Urban & Rural	UMIC	Multiple (x2) 24hR	Bulgarian	Yes	EFSA & GDD*§	NA	Completed
BRA	Pesquisa de Orçamentos Familiares^(37)^	2008–2009	National	Yes	34 003	10–104	M & F	Urban & Rural	UMIC	Multiple (x2) 24hR	Portuguese	NA	FAO	NA	Not completed
BRA	Brazilian Study of Cardiovascular Risks in Adolescents (ERICA)^(38)^	2013–2014	National	No	71 971	12–17	M & F	Urban & Rural	UMIC	Multiple (x2) 24hR	Portuguese	Yes	GDD	A	Completed
CAN	Canadian Community Health Survey (CCHS)^(39)^	2015	National	Yes	20 483	1–95	M & F	Urban & Rural	HIC	Multiple (x2) 24hR	English, French	NA	GDD	C	Completed
COD	Women First Preconception Maternal Nutrition RCT^(40)^	2014–2016	Community level	No	214	16–34	F	Rural	LIC	Multiple (x2) 24hR	French	NA	FAO	NA	Completed
DEU	DOrtmund Nutritional and Anthropometric Longitudinally Designed Study (DONALD)^(41)^	2006–2008	Community level	Yes	921	1–10	M & F	Urban	HIC	Multiple (up to 3) FR	German	Yes	EFSA & GDD*§	NA	Completed
DEU	DOrtmund Nutritional and Anthropometric Longitudinally Designed Study (DONALD)^(42)^	2015–2019	Community level	Yes	559	0·94–19·2	M & F	Urban	HIC	Multiple (up to 6) FR	German	Yes	GDD	A	Completed
ECU	Encuesta Nacional de Salud y Nutrición^12^ (ENSANUT-ECU)^(43)^	2012	National	Yes	92 502	0–60	M & F	Urban & Rural	UMIC	Single 24hR	Spanish	No	GDD	C	Completed
ESP	Hábitos dietéticos y estado nutricional de la población española infantil y juvenil*¶ (Estudio enKid)^(44)^	1998–2000	National	No	382	1–14	M & F	Urban & Rural	HIC	Multiple (x2) 24hR	Spanish	No	EFSA	NA	Completed
EST	National Dietary Survey (The Baltic Project) (RTU)^(45)^	1997	National	Yes	1866	16–64	M & F	Urban & Rural	HIC	Single 24hR	Estonian, Russian	Yes	EFSA & GDD*§	NA	Completed
EST	National Dietary Survey (RTU)^(46)^	2013–2015	National	Yes	4647	0·25–75	M & F	Urban & Rural	HIC	Multiple (x2) 24hR or FR	Estonian, Russian	Yes	EFSA & GDD*§	NA	Completed
ETH	Dietary behavior, food and nutrient intake of women during pregnancy in Southern Ethiopia^(47)^	2013	Sub-national	No	323	18–45	F	Rural	LIC	Multiple (x2) 24hR	Amharic	Yes	GDD	A	Completed
ETH	Dietary practices, maternal nutritional status and child stunting in pulse and non-pulse growing rural communities^(48)^	2013	Sub-national	No	217 217	0·25–5 16–48	M & F	Rural	LIC	Single weighed FR	Amharic, English	Yes	GDD	A	Completed
FIN	Increased health and well-being in preschools (DAGIS) cross-sectional survey^(49)^	2015–2016	Sub-national	No	864	3–6	M & F	Urban & Rural	HIC	Multiple (up to 5) FR	Finnish	Yes	GDD	A	Completed
GTM	Women First Preconception Maternal Nutrition RCT^(50)^	2014–2016	Community level	No	222	17–33	F	Rural	UMIC	Multiple (x2) 24hR	Spanish	NA	FAO	NA	Completed
IND	Diet and nutritional status of rural population, prevalence of hypertension and diabetes among adults, and infant and young child feeding practices^(51)^	2009–2012	National	No	39 719	0·5–99	M & F	Rural	LMIC	Single 24hR	English	No	GDD	A	Completed
IND	Women First Preconception Maternal Nutrition RCT^(52)^	2014–2015	Community level	No	242	16–33	F	Rural	LMIC	Multiple (x2) 24hR	Kannada	NA	FAO	NA	Completed
IRN	Isfahan Healthy Heart Program (IHHP)^(53)^	2007	Sub-national	No	1342	19–85	M & F	Urban	UMIC	Single 24hR	Persian	No	GDD	B	Completed
ISR	Mabat First Israeli National Health and Nutrition Survey^(54)^	1999–2001	National	Yes	3242	25–64	M & F	Urban & Rural	HIC	Single 24hR	Hebrew, Russian, Arabic	No	GDD	B	Completed
ISR	Mabat Youth - First Israeli National Health and Nutrition Survey in 7–12^th^ grade students^(55)^	2003–2004	National	Yes	578	12–18	M & F	Urban & Rural	HIC	Single 24hR	Hebrew, Russian, Arabic	No	GDD	B	Completed
ISR	Mabat Zahav - National Health and Nutrition Survey in ages 65 and over^(56)^	2005–2006	National	Yes	1786	65–96	M & F	Urban	HIC	Single 24hR	Hebrew, Russian, Arabic	No	GDD	B	Completed
ITA	Nationwide Food Consumption Study in Italy (INRAN-SCAI)^(57)^	2005–2006	National	Yes	3323	0·1–97	M & F	Urban & Rural	HIC	Multiple (x3) FR	Italian	No	EFSA	NA	Completed
KEN	Kenya National Micronutrient Survey (KNMS)^(58)^	2011	National	Yes	277 549	0·5–4·9 15–49	M & F F	Urban & Rural	LMIC	Multiple (x2) 24hR	English	Yes	GDD	A	Completed
KEN	Improving Nutrition through Local Agrobiodiversity Survey (INULA study)^(59)^	2012–2014	Sub-national	No	881	0·46–65·3	F	Rural	LMIC	Multiple (x2) 24hR	Kiswahili, English	NA	FAO	NA	Completed
KEN	Innovative, participatory tools for dietary assessment and nutrition education in Turkana County^(60)^	2016	Sub-national	No	425	0·58–52	M & F	Rural	LMIC	Multiple (x2) 24hR	Kiswahili, Turkana, English	NA	FAO	NA	Completed
KEN	Diverse seeds to support on-farm biodiversity for healthy people in resilient landscapes: Baseline Survey^(61)^	2018	Sub-national	No	741	0·5–59	M & F	Rural	LMIC	Multiple (x2) 24hR	Kiswahili, English	NA	FAO	NA	Completed
KOR	Korea National Health and Nutrition Examination Survey (KNHANES)^(62)^	2017	National	Yes	7167	0·6–80	M & F	Urban & Rural	HIC	Single 24hR	Korean	No	GDD	A	Completed
LAO	Lao Food Consumption Survey^(63)^	2016–2017	National	No	2045	0·25–89	M & F	Urban & Rural	LMIC	Single 24hR	Lao	NA	FAO	NA	Completed
MEX	National Health and Nutrition Survey (ENSANUT)^(64)^	2012	National	Yes	10 685	0·04–104	M & F	Urban & Rural	UMIC	Multiple (x2) 24hR	Spanish	Yes	GDD	A	Completed
MOZ	Estudo do Estado Nutricional e da Dieta em Raparigas Adolescentes na Zambézia (ZANE Study)^(65)^	2010	Sub-national	No	551	15–18	F	Urban & Rural	LIC	Multiple (up to 3) 24hR	English	Yes	GDD	A	Completed
MYS	Malaysia Lipid Study^(66)^	2012–2013	Sub- national	No	577	20–67	M & F	Urban	UMIC	Multiple (x3) 24hR	Bahasa Malaysia, Chinese, Tamil, English	Yes	GDD	B	Completed
PAK	Women First Preconception Maternal Nutrition RCT^(67)^	2014–2016	Community level	No	270	16–35	F	Rural	LMIC	Multiple (x2) 24hR	Sindhi	NA	FAO	NA	Completed
PHL	Cebu Longitudinal Health and Nutrition Survey*¶ (CLHNS)^(68)^	2002	Sub-national	Yes	4153	17–66	M & F	Urban & Rural	LMIC	Multiple (x2) 24hR	Pilipino*||	NA	GDD	C	Completed
PHL	6th National Nutrition Survey^(69)^	2003–2004	National	Yes	1205	15–47	F	Urban	LMIC	Multiple (x2) 24hR	Pilipino, English	NA	FAO	NA	Completed
PHL	Cebu Longitudinal Health and Nutrition Survey*¶ (CLHNS)^(70)^	2005	Sub-national	Yes	3930	20–69	M & F	Urban & Rural	LMIC	Multiple (x2) 24hR	Pilipino*||	NA	GDD	C	Completed
PRT	National food, nutrition and physical activity survey of the Portuguese general population (IAN-AF)^(71)^	2015–2016	National	Yes	6553	0·25–84	M & F	Urban & Rural	HIC	Multiple (x2) 24hR or FR	Portuguese	No	EFSA & GDD*§	NA	Completed
PRT	National food and physical activity survey (IAN-AF) - Pregnant women^(71)^	2015–2016	National	No	166	17–46	F	Urban & Rural	HIC	Multiple (x2) 24hR	Portuguese	No	EFSA & GDD*§	NA	Completed
ROU	DIETA PILOT Adults^(72)^	2012	National	No	1382	19–92	M & F	Urban & Rural	HIC	Multiple (x7) FR	Romanian	Yes	EFSA & GDD*§	NA	Completed
ROU	DIETA PILOT Children^(72)^	2012	National	No	773	3–18	M & F	Urban & Rural	HIC	Single 24hR	Romanian	Yes	EFSA & GDD*§	NA	Completed
SVK	Compilation of existing individual food consumption data collected within the most recent national dietary surveys in Europe (SK MON)^(73)^	2008	National	No	2761	17–68	M & F	Urban & Rural	HIC	Single 24hR	Slovak	No	EFSA	NA	Completed
SWE	Swedish National Dietary Survey - Riksmaten^(74)^	1997–1998	National	Yes	1210	17–79	M & F	Urban & Rural	HIC	Multiple (x7) FR	Swedish	No	EFSA & GDD*§	NA	Completed
SWE	Swedish National Dietary Survey in Children – Riksmaten barn^(75)^	2003	National	Yes	2491	3–13	M & F	Urban & Rural	HIC	Multiple (x4) FR	Swedish	No	EFSA & GDD*§	NA	Completed
SWE	Swedish National Dietary Survey – Riksmaten vuxna^(76)^	2010–2011	National	Yes	1797	18–80	M & F	Urban & Rural	HIC	Multiple (x4) FR	Swedish	No	EFSA & GDD*§	NA	Completed
TWN	Nutrition and Health Survey in Taiwan (NAHSIT)^(77)^	2005–2008	National	Yes	Unknown	0–6, 19+	M & F	Urban & Rural	HIC	Multiple (x2) 24hR*‡	Chinese	Yes	GDD	A	Not completed
TZA	Scale_N Nutrition Survey^(78)^	2016	Subnational	No	1332	5–75	F	Rural	LMIC	Single 24hR		NA	FAO	NA	Completed
UGA	HarvestPlus Reaching End Users (REU) Orange-Fleshed Sweet Potato (OFSP) Project^(79)^	2007–2008	Sub-national	No	577	13–82	F	Rural	LIC	Multiple (x2) 24hR	English	NA	FAO	NA	Completed
USA	National Health and Nutrition Examination Survey (NHANES)^(80)^	2013–2014	National	Yes	8661	0–80	M & F	Urban & Rural	HIC	Multiple (x2) 24hR	English	NA	GDD	C	Completed
USA	National Health and Nutrition Examination Survey (NHANES)^(81)^	2015–2016	National	Yes	8506	0–80	M & F	Urban & Rural	HIC	Multiple (x2) 24hR	English	NA	GDD	C	Completed
USA	National Health and Nutrition Examination Survey (NHANES)^(82)^	2017–2018	National	Yes	7641	0–80	M & F	Urban & Rural	HIC	Multiple (x2) 24hR	English	NA	GDD	C	Not completed
ZMB	Food consumption and Vitamin A status survey^(83)^	2009	Sub-national	No	955 710	0–6 16–70	M & F F	Rural	LMIC	Multiple (x2) 24hR	English	NA	FAO	NA	Completed

12hR, 12-h recall; 24hR, 24-h recall; ARG, Argentina; BGD, Bangladesh; BRA, Brazil; BGR, Bulgaria; BFA, Burkina Faso; CAN, Canada; COD, Democratic Republic of the Congo; DEU, Germany; EFSA, European Food Safety Authority; ECU, Ecuador; ESP, Spain; EST, Estonia; ETH, Ethiopia; F, female; FIN, Finland; FR, food record; GDD, Global Dietary Database; GTM, Guatemala; IND, India; IRN, Iran, Islamic Republic of; ISR, Israel; ITA, Italy; KEN, Kenya; KOR, Republic of Korea; LAO, Lao People’s Democratic Republic; M, male; MEX, Mexico; MOZ, Mozambique; MYS, Malaysia; PAK, Pakistan; PHL, Philippines; PRT, Portugal; ROU, Romania; SVK, Slovakia; SWE, Sweden; TWN, Taiwan; TZA, Tanzania; UGA, Uganda; USA, United States of America; ZMB, Zambia.

* As sub-nationals were considered the surveys which were representative of a major region of the country and as community-level the surveys which were representative of a single city, town or village.

† Rolling programs refer to surveys whose administration is repeated every year or every few years; these surveys may or may not follow the same population.

‡ The reported sample size, age range and sex refer to the participants with available dietary data and not to the overall survey sample.

§ Country-income level was based on the World Bank classification of countries into low-income (LIC), lower-middle income (LMIC), upper-middle income (UMIC) and high-income (HIC) countries.^(22)^.

|| The number in the parenthesis is the number of recalls or records collected per participant. When multiple recall/ record is reported, it applies to either part of the sample or all participants.

¶ Financial support was offered to the data owners of all surveys, with the exception of publicly available datasets and surveys whose harmonisation had been already completed by FAO.

** The leading initiative was responsible for harmonising the dietary data or for guiding the data owners to perform the harmonisation themselves.

*† Refers to the data sharing option selected by data owners, and it is only applicable to surveys whose harmonisation was led by GDD. Option A refers to data owners agreeing to structure their data per the GDD requirements and perform the FoodEx2 mapping; option B includes agreement to data structure only and GDD to perform the FoodEx2 mapping and option C refers to sharing data as is, without structure or FoodEx2 mapping and GDD performs the overall harmonisation. NA refers to surveys that have been harmonised by FAO or EFSA.

*‡ Refers to the harmonisation status of each survey. Three of the fifty-five surveys that were included for harmonisation were not harmonised due to time and resources restrictions.

*§ EFSA oversaw the data structure and FoodEx2 mapping and GDD the addition of food matching.

*|| Data were collected in the country language (Bengali or Pilipino), but the English version of the datasets was also available in the original public source.

*¶ Food matching is missing. The nutrient content for the reported food items was not allowed to be made publicly available. For Spain, food matching was not performed due to the COVID-19 pandemic.

### Data harmonisation

For the twenty-four surveys retrieved from data owners, eleven agreed to provide both data structure (including mixed dish disaggregation and food matching) and FoodEx2 mapping; five agreed to data structure only and the GDD performed the FoodEx2 mapping and eight provided their data as is, including seven publicly available ones, which the GDD fully harmonised (Table 2). For fifteen surveys retrieved through EFSA that were lacking the food matching, thirteen agreed to perform this following contact by the GDD. All sixteen surveys retrieved through the FAO/WHO GIFT platform were fully harmonised. Among data owners performing the harmonisation, the most resource-intensive action was mixed dish disaggregation (generally 2–6 months to complete), followed by FoodEx2 mapping (1–4 months) and food matching (1–3 months). With experience, such as in the GDD investigator team working with surveys over time, the harmonisation of each public dataset took from 1 to 3 months. Common challenges included understanding the reported information and data, especially related to variable dataset documentation, and FoodEx2 mapping for highly localised food items.

**Table 2:** Survey and dietary data characteristics of the included 24-h recall/record dietary surveys by country income category

	**Overall** (*n* 55)	**HIC** (*n* 24)	**UMIC** (*n* 10)	**LMIC** (*n* 15)	**LIC** (*n* 6)
Representativeness					
National	32	21	6	5	0
Sub-national	15	1	2	7	5
Community level	8	2	2	3	1
Rolling program	27	18	4	5	0
Dietary assessment method*					
Single 24-h recall	16	7	4	5	0
Single food record	2	0	0	1	1
Multiple 24-h recall	30	9	6	10	5
Multiple food record	10	10	0	0	0
Survey administration method					
Computer-assisted interview	10	7	3	0	0
Pen and paper	44	17	7	15	5
Combination of the two	1	0	0	0	1
Institutional home (Data owner)†					
Governmental institution	22	14	5	3	0
Academic institution	22	9	4	7	2
Other institution	9	1	1	5	2
Physical person	2	0	0	0	2
Harmonisation option selected‡					
Structure and FoodEx2 mapping	11	4	2	2	3
Only data structure	5	3	2	0	0
Data shared as is	8	4	1	3	0
Mixed dish disaggregation available before harmonisation‡	12	8	2	2	0
Original FCDS, before FoodEx2					
None	39	15	5	13	6
National/ Survey-specific/ Software	17	9	5	2	0
Unique food items, median (IQR)§					
Median	719	1602	717	269	149
IQR	232–1804	1068–2784	458–1096	87–684	92–184
Nutrients available in dataset§					
Median	26	29	31	17	17
IQR	17–31	27–32	18–31	14–21	16–19

HIC, high-income countries; LMIC, lower-middle income countries; LIC, low-income countries; UMIC, upper-middle income countries; FCDS, food classification and description system; IQR, interquartile range.

* The sum of surveys across the different assessment methods is more than the overall sum because three surveys used both 24-h recall and food record.

† The institutional home refers to the data owning institution/organisation of the survey data. The data owning institution could be either governmental (e.g. ministry, statistical agency), an academic institution (e.g. university), other institution (e.g. non-governmental organisations, independent research institutes), or individuals who self-funded the survey data collection.

‡ This applies only to the twenty-four GDD surveys. The surveys the GDD received from FAO and EFSA were already harmonised.

§ Values refer to the harmonised – not to the original – dataset. Only surveys whose harmonisation was complete (*n* 52 of fifty-five available) were used.

### Survey characteristics

**Fig. 3:**
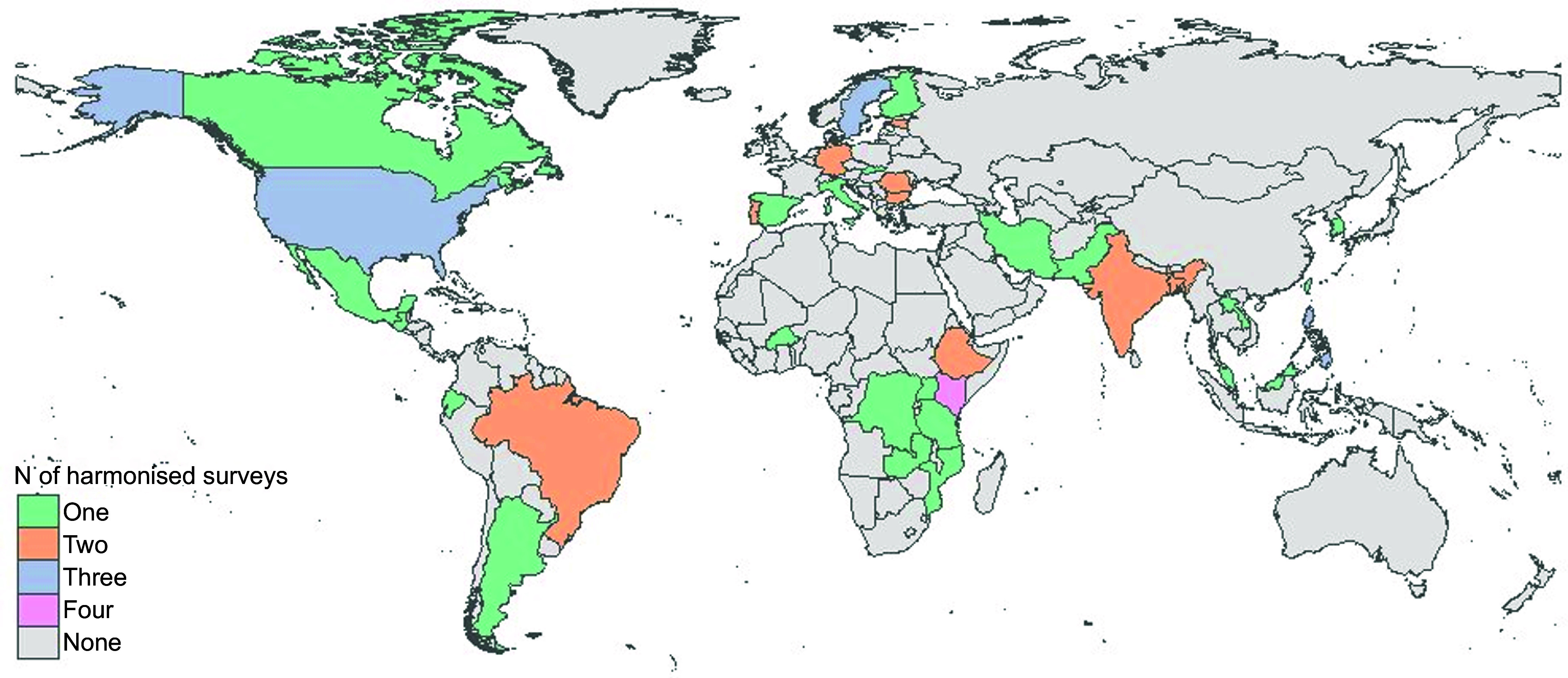
Availability of harmonised dietary surveys per country. The map presents the 55 surveys, from 35 countries, that have agreed to the GDD harmonisation. Of these, the harmonisation has not been completed for three surveys (one in Brazil, one in Taiwan and one in the USA) due to time and resource restrictions

The fifty-five surveys included for harmonisation were from thirty-five countries (Fig. 3) and included twenty-four surveys from HIC, ten from UMIC, fifteen from LMIC and six from LIC (Table 1). Most surveys (*n* 32, 58 %) were nationally representative, fifteen (27 %) were sub-national, and eight (15 %) were at the community level, especially in LIC and LMIC where nationally representative 24-h recall data were limited (Table 2). Half of the surveys (*n* 27) were rolling programs. About two-thirds (*n* 34, 62 %) were performed after 2010 and few (*n* 2, 4 %) before 2000. The predominant diet assessment method was 24-h recall (*n* 43, 78 %), followed by food records (*n* 9, 16 %), whereas three (5 %) surveys used both methods. Most surveys (*n* 38, 69 %) collected multiple recall/ record data for a subset or all participants. Generally, LIC and LMIC had smaller-scale surveys (median 826 participants; IQR 434–1665), compared with HIC and UMIC (1832; 893–6860). Most surveys (*n* 44, 80 %) collected data for both sexes; most (*n* 49, 89 %), on children and adolescents (ages 0–19 years), including twenty-seven surveys with data on children under 5 years of age; and nearly two-thirds (*n* 32, 58 %), from both rural and urban areas. Twelve of the twenty-one surveys from LIC and LMIC were administered in children under 5 years of age and/or women of reproductive age. Data reporting language varied across and within datasets (twenty-eight languages and multiple dialects). Surveys were originally collected using pen and paper in forty-four surveys (80 %), with computer-assisted interviews only in HIC and UMIC. Survey data owners varied between governmental institutions (*n* 22), academic institutions (*n* 22), independent research or non-governmental organisations (*n* 9) and individual scientists (*n* 2).

### Survey data

**Fig. 4.**
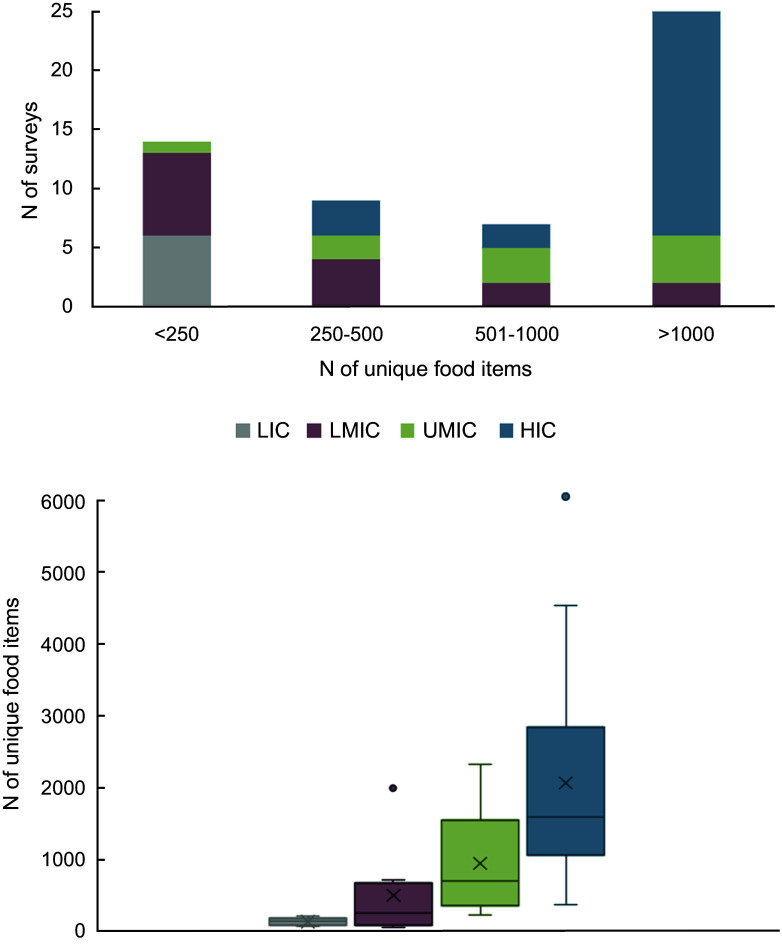
Number of unique food items per dietary survey by country income level. The *top panel* shows the distribution of surveys from low-income (LIC), lower-middle income (LMIC), upper-middle income (UMIC), and high-income countries (HIC) across 4 range categories of unique food items per survey. All 55 surveys included for harmonisation were used. The *bottom panel* shows the absolute number of unique food items per survey grouped by country income level. Boxplots are shown, with horizontal lines representing the median value; shaded bars representing the interquartile range (IQR); whiskers representing maximum (top) and minimum (bottom) value excluding outliers, x’s representing the mean value and circles representing outliers. Only surveys whose harmonisation was complete were included (*n* 52 of 55)

Surveys originally had either no (*n* 39) or a national or survey-specific (*n* 16) food classification system in place. The number of reported unique food items varied across datasets (median 719, IQR 232–1804, max 6063) (Fig. 4). All LIC surveys had less than 250 unique food items (149, 92–184); LMIC (269, 87–684) and UMIC (717, 458–1096) surveys included, in general, less than 1000 food items; and HIC surveys generally included over 1000 unique food items (1602, 1068–2784). Mixed dish disaggregation was originally available for twelve of the twenty-four surveys retrieved from data owners; for ten, disaggregation was performed as part of the harmonisation; for two publicly available surveys, this information was not available. For eight datasets, the food description remained in the original language, because FoodEx2 mapping, which enables translation into English, was performed by data owners.

All datasets captured the whole diet; more than half (*n* 31) further captured water consumption. Food matching in HIC and UMIC relied mainly on national FCT/FCDB (twenty-five of twenty-nine surveys with nutrient intakes reported) that contained a large number of available foods (1626, 1040–4300) and nutrients. LIC and LMIC relied also primarily on local national FCT (fourteen of eighteen surveys with nutrient intakes reported), but with a lower number of available food items (343, 152–696) and nutrients; to incorporate additional nutrients into their dataset, data owners generally attempted to use large FCDB of HIC, such as the USDA Food Database (the metadata information that is published as part of the harmonised data includes the specific FCT/FCDB used for each survey).

**Fig. 5:**
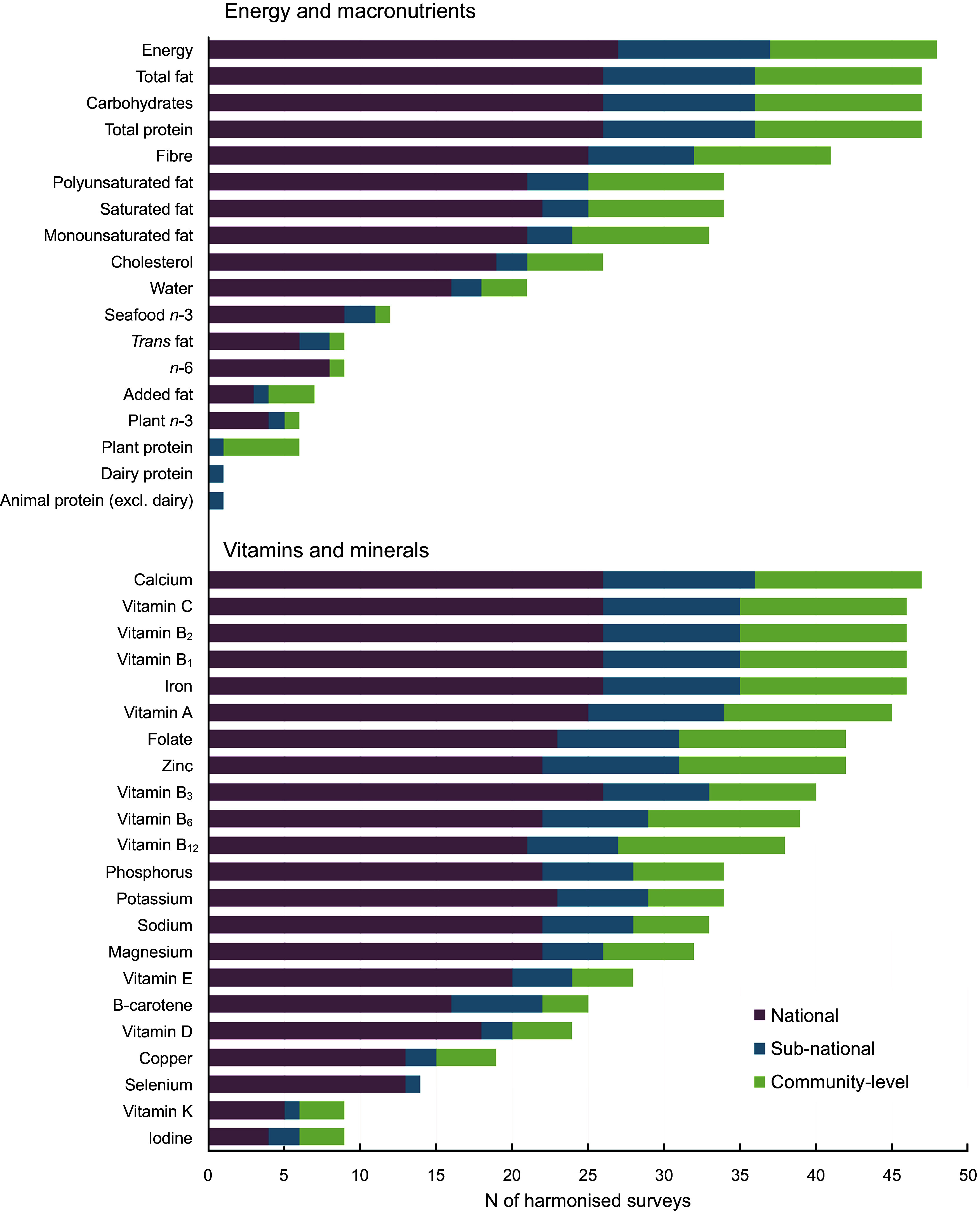
Availability of energy and nutrients across harmonised dietary datasets, by survey representativeness. Only surveys whose harmonisation has been completed and they report nutrient intakes are presented (*n* 48 of 55). The figure presents the 40 nutrients requested; datasets may contain additional nutrients (e.g. total sucrose, retinol) that are not listed here

Four surveys only reported on foods, of which three publicly available ones did not permit the nutrient composition to be made public and 1 did not manage to add the food matching due to constraints related to the COVID-19 pandemic. In the remaining forty-eight surveys, twenty-four (sd: ±8) nutrients were available on average per dataset (Table 2), with generally fewer available in LIC and LMIC (19, ±5) *v*. in HIC and UMIC (28, ±7). Energy was available in all forty-eight datasets reporting nutrient intakes, followed by total protein, carbohydrates and total fat (each *N* 47; Fig.5). The most frequently reported vitamins and minerals were Ca (*n* 47), Fe (*n* 46), vitamin B_1_ (*n* 46), vitamin B_2_ (*n* 46) and vitamin C (*n* 46) and the least, vitamin K (*n* 9) and iodine (*n* 9). Other less frequently reported dietary factors included protein subtypes (*n* 1–6 surveys) and plant *n*-3 fatty acids (*n* 6).

### Public dissemination

As of October 2023, the final output of the harmonisation process are fifty-two surveys, which have been harmonised and made publicly available and free to download through the GDD website (www.globaldietarydatabase.org)^(23)^; twenty seven of these are also available through the FAO/WHO GIFT website (www.fao.org/gift-individual-food-consumption)^(21)^.

## Discussion

This investigation reports on the process and results for identification, retrieval, harmonisation and public dissemination of individual-level dietary data at their finest level from dietary surveys around the world. The GDD identified and included for harmonisation fifty-five surveys – fifty two were ultimately harmonised – using 24-h recalls (78 %), food records (16 %) or both (5 %) from thirty-five countries. Most surveys (58 %) were nationally representative, and countries of all income levels were represented. The surveys largely captured both sexes and diverse ages from birth to late in life and generally collected data for both rural and urban areas. Notably, the majority of these surveys (76 %) were not previously publicly available, requiring extensive work to contact data owning institutions, access data and complete data sharing agreements for public dissemination. Thus far, these harmonised surveys represent the most comprehensive collection of comparable, standardised dietary data at their most granular level globally.

Survey methods for collecting and reporting data were variable, including dataset structure, reporting language, available level of detail in recorded foods and use of an FCDS. This meant that data harmonisation and analysis from diverse countries were particularly complex undertakings. For example, reporting language included over twenty-eight languages and dialects across the fifty-five datasets, stressing the importance of harmonisation to a common language and terminology across datasets. In addition, the combination of open-ended questions in a 24-h recall or record plus local food names resulted in similar items being reported differently across datasets and with varying detail (e.g. ‘grilled beef’ *v*. ‘steak, cow, barbequed’). Extensive efforts and clarifications by data owners were often required to fully understand the foods reported. Many surveys had not previously disaggregated their mixed dishes, preventing valid estimates of intakes of different food groups (e.g. fruits, vegetables and meats). Finally, most surveys had not used an FCDS prior to this effort, and those that did used a local or survey-specific one, not comparable across datasets. This effort demonstrated that such foundational challenges can be addressed with a coordinated effort to harmonise and share the original survey information and it greatly advanced data completeness, comparability and availability.

The availability of nutrient estimates following harmonisation and comprehensiveness of the FCT used also varied substantially. In general, HIC and UMIC reported more nutrients than LIC and LMIC (median 30 *v*. 17). LIC and LMIC typically focused on energy, total protein, carbohydrates, total fat and key vitamins and minerals relevant to specific micronutrient deficiencies in maternal and child health, such as Ca, Fe, Zn and vitamins A, B1, B2 and C. These surveys rarely reported nutrients relevant to diet-related chronic diseases, such as total polyunsaturated fat, plant or seafood *n*-3 fat, *trans* fat, Na or potassium. FCT were also typically more comprehensive in HIC and UMIC than in LIC and LMIC (median ∼1626 *v*. ∼343 food items). This partly reflects the greater diversity of available food items commonly consumed in high-income nations, such as specific packaged and processed foods, compared with low-income nations where staples and variety are much smaller. However, this also reflects less available local food composition data in middle- and low-income nations. Given the societal burdens of malnutrition in all its forms in every country worldwide, including diet-related chronic diseases^(13)^, our findings highlight the need for more comprehensive nutrient assessments in LIC and LMIC worldwide, coupled with investment in expanded local FCT.

Our extensive searches only identified twenty-one 24-h recall or record surveys from LIC and LMIC available for inclusion in this effort. These surveys were usually administered within a specific region or community; were small-scale and aimed to evaluate specific nutrients and, as such, nutrient deficiencies, especially focused on maternal and young child nutrition. Such surveys also most often collected data via pen and paper, raising challenges for standardised data collection and future data manipulation^(30)^. Half of the included surveys (49 %) were rolling programs, i.e. with planned additional cycles of dietary assessment. This raises the possibility of far more efficient, or even prospective, data harmonisation and FoodEx2 mapping in future survey cycles. However, most rolling programs were in HIC or UMIC (*n* 22), fewer were in LMIC (*n* 5), and none were identified in LIC. These findings stress such disparities in dietary data availability and quality across countries of different income levels, emphasizing the need for substantial new investments in dietary surveillance and harmonisation efforts in LIC. For example, ongoing major investments in supplementation programs, crop biofortification and food fortification will be more inefficient and less effective without reliable, valid surveillance data on the specific nutrient gaps in specific population subgroups and the specific food intakes that should be targets of fortification delivery intakes.

The current investigation has several strengths. Systematic searches were performed across multiple online databases, complemented by extensive contacts with data owners and nutrition experts worldwide to identify surveys with valid 24-h recall or food record data. The collaboration with FAO and EFSA advanced global coordination including sharing of expertise, experiences, methodological approaches and networks. Standardised methods, protocols and materials were developed for all steps of this effort in order to reduce the likelihood of error during data acquisition and achieve high-quality harmonisation. Individual-level dietary data at their finest level, as well as detailed socio-demographic information per participant, were collected and harmonised, which altogether capture the overall diet of the population, minimise errors in reporting and analysis and provide critical information on dietary heterogeneity within countries and populations. A common FCDS, FoodEx2, was used to address the challenge of food description and classification variations, improve data interpretability, allow for detailed analysis and enable comparability of estimated diet intakes within and between nations, further overcoming any language barriers. To ensure data integrity and maximise internal and external consistency, data owners were extensively trained on restructuring and harmonizing their datasets using FoodEx2 and rigorous data checks throughout the harmonisation process were performed. The outcome of this process, the harmonisation methods and final datasets were made publicly available and freely accessible. These can serve as a critical public resource for researchers, health agencies and governments to inform future dietary data collection efforts and promote global collaboration and capacity development. Such timely, reliable and comparable nutrition methods and data can be leveraged to understand, quantify and address the corresponding nutrition burden locally, nationally and across the world.

Potential limitations should be considered. Nationally representative 24-h recall/record data were less common in LIC and LMIC, requiring greater reliance on subnational surveys. Variability in primary data collection methods and details, as expected, precluded collecting all variables of interest, such as socio-demographics, dietary information and nutrients and leveraging all FoodEx2 facets for every food item. However, by using the rigorous protocols and materials developed, all available information in the primary datasets were converted or derived and standardised. While ultimately fifty-five surveys were included by the GDD for harmonisation, not all potentially eligible data owning institutions were able to participate. Interestingly, the majority of exclusions were due to non-response, no final decision, unwillingness to share their data, with only a few noting workload or resource challenges. This raises the question of whether strengthened global coordination, including prioritisation from national governments, could significantly increase such dietary harmonisation efforts with additional resource investment.

In summary, this effort demonstrates that new investments in institutional support and training can result in diverse global 24-h dietary recalls/records being harmonised to provide highly granular, standardised and publicly available dietary data from around the world. Such high-quality comparable data can promote and support nutrition programming, research and capacity development worldwide. This work also identifies key gaps in 24-h recall data collection, facilitates and promotes country capacity development that can alleviate many of the barriers that prevent routine harmonised collection of 24-h recall data and highlights the importance of collecting comprehensive harmonised dietary data systematically in all nations.

## Supporting information

Karageorgou et al. supplementary material Karageorgou et al. supplementary material

## The Global Dietary Database harmonisation collaborators

Ute Alexy, University of Bonn; Jennifer Ali, Statistics Canada; Lilian Aluso, Consultative Group on International Agricultural Research; Rosangela Alves Pereira, Federal University of Rio de Janeiro; Imelda Angeles Agdeppa, Food and Nutrition Research Institute; Joanne E. Arsenault, University of California Davis; Laxmaiah Avula, ICMR-National Institute of Nutrition; Donka Baykova, Medical Center Markovs; Julia Boedecker, Consultative Group on International Agricultural Research; Ken Brown, University of California Davis; Zipporah Bukania, Kenya Medical Research Institute; Mario Capanzana, Food and Nutrition Research Institute; Hsing-Yi Chang, National Health Research Institutes; Justin Chileshe, Tropical Diseases Research Center; Alan de Brauw, International Food Policy Research Institute; Stefaan De Henaw, Ghent University; Amanda de Moura Souza, Universidad Federal de Rio de Janeiro; Josie Desnacido, Food and Nutrition Research Institute; Tshilenge S. Diba, University of Colorado; Daovieng Douangvichit, Lao Tropical and Public Health Institute; Vesselka Duleva, National Centre of Public Health and Analyses; Laila Eleraky, University of Hohenheim; Maijaliisa Erkkola, University of Helsinki; Getahun Ersino, University of Saskatchewan; Riitta Freese, University of Helsinki; Rebecca Goldsmith, Israel Center for Disease Control; Ron Gravel, Statistics Canada; K. Michael Hambidge, University of Colorado; Carol Henry, University of Saskatchewan; Christine Hotz; Meshram Indrapal, ICMR-National Institute of Nutrition; Veronika Janská, Public Health Authority of the Slovak Republic; Irmgard Jordan, Justus Liebig University Giessen; Tilakavati Karupaiah, Taylor’s University; Gudrun B. Keding, University of Goettingen; Lital Keinan-Boker, Israel Center for Disease Control; Umber S Khan, University of Colorado; Yeri Kombe, Kenya Medical Research Institute; Liisa M Korkalo, University of Helsinki; Nancy Krebs, University of Colorado; Maarja Kukk, National Institute for Health Development; Sanghui Kweon, Korea Centers for Disease Control and Prevention; Carl Lachat, Ghent University; Anna Karin Lindroos, National Food Agency; Julie Long, University of Colorado; Carla Lopes, Institute of Public Health - University of Porto; Chanthaly Luangphaxay, Lao Tropical and Public Health Institute; Maryam Maghroun, Isfahan Cardiovascular Research Institute; Yves Martin-Prevel, Institut de Recherche pour le Développement; Shivanand C. Mastiholi, University of Colorado; J.V. Meenakshi, University of Delhi; Noushin Mohammadifard, Isfahan Cardiovascular Research Institute; Carlos Monteiro, University of Sao Paulo; Lotta Moraeus, National Food Agency; Mourad Moursi, International Food Policy Research Institute; Moses Mwangi, Kenya Medical Research Institute; Sydney Mwanza, Tropical Diseases Research Center; Keiu Nelis, National Institute for Health Development; Anca Nicolau, Dunărea de Jos University of Galaţi; Laetitia Nikiema, WHO; Arlappa Nimmathota, ICMR-National Institute of Nutrition; Ute Nothlings, University of Bonn; Eha Nurk, National Institute for Health Development; Francis Oduor, Consultative Group on International Agricultural Research; Kyungwon Oh, Korea Centers for Disease Control and Prevention; Wen-Harn Pan, Academia Sinica; Myungsook Park, Korea Centers for Disease Control and Prevention; Jessica Petrelius Sipinen, National Food Agency; Stefka Petrova, National Centre of Public Health and Analyses; Raffaela Piccinelli, CREA-Alimenti e Nutrizione; Harikumar Rachakulla, ICMR-National Institute of Nutrition; Hemalatha Rajkumar, ICMR-National Institute of Nutrition; Ivonne Ramirez Silva, Instituto Nacional de Salud Publica; Lalka Rangelova, National Centre of Public Health and Analyses; Lourdes Ribas-Barba, Fundación para la Investigación Nutricional; Juan Rivera, Instituto Nacional de Salud Publica; Sonia Rodriguez Ramirez, Instituto Nacional de Salud Publica; Eva Roos, University of Helsinki; Alicia Rovirosa, Centro de Estudios sobre Nutrición Infantil; Constance Rybak, Leibniz Centre for Agricultural Landscape Research; Nizal Sarrafzadegan, Isfahan Cardiovascular Research Institute; Lluis Serra-Majem, University of Las Palmas de Gran Canaria; Stefania Sette, CREA-Alimenti e Nutrizione; Milton Severo, Institute of Public Health - University of Porto; Tal Shimony, Israel Center for Disease Control; Ward Siamusantu, National Food and Nutrition Commission; Rosely Sichieri, Universidade do Estado do Rio de Janeiro; Latsamy Siengsounthone, Lao Tropical and Public Health Institute; Wolfgang Stuetz, University of Hohenheim; Kalyana Sundram, Malaysian Palm Oil Council; Tina Tao, Statistics Canada; Tamene Taye Asayehu, Addis Ababa Science and Technology University; Gabriela Tejeda, University of Colorado; Celine Termote, Alliance of Bioversity International and CIAT; Duarte Torres, University of Porto; Cornelia Tudorie, Dunărea de Jos University of Galaţi; Aida Turrini, CREA-Alimenti e Nutrizione; Sirje Vaask, Tallinn University; Eva Warensjö Lemming, National Food Agency; Lydiah M. Waswa, University of Goettingen; Elizabeth Yakes-Jimenez, University of New Mexico; Maria Elisa Zapata, Universidad del Centro Educativo Latinoamericano; Gordon Zello, University of Saskatchewan; Corina Aurelia Zugravu, University of Medicine and Pharmacy Carol Davila.

## Supplementary material

For supplementary material accompanying this paper visit https://doi.org/10.1017/S1368980024000211
