# Supplementary material for: Harmonising dietary datasets for global surveillance: methods and findings from the Global Dietary Database
Source: Public Health Nutr. 2024 Jan 19;27(1):e47. doi: 10.1017/S1368980024000211 (PMC10882534; doi:10.1017/S1368980024000211)
Supplement: Karageorgou et al. supplementary material [file S1368980024000211sup001.docx]

**SUPPLEMENT**

**Table of Contents**

[**Table S1.** Materials developed to support the GDD harmonization process.^1^ 2](#_Toc152628071)

[**Table S2.** Criteria for determining survey eligibility for the GDD dietary data harmonization. 4](#_Toc152628072)

[**Table S3.** Criteria for prioritizing eligible surveys for the GDD dietary data harmonization. 5](#_Toc152628073)

[**Table S4.** Variable definitions and values for the survey participant data (Participant Codebook). 6](#_Toc152628074)

[**Table S5.** Variable definitions and values for the dietary data (Diet Codebook). 9](#_Toc152628075)

[**Table S6.** Top-level food groups of the FoodEx2 classification system. 15](#_Toc152628076)

[**Table S7.** The facets of the FoodEx2 description system. 16](#_Toc152628077)

[**Figure S1.** The Global Dietary Database contact process flowchart. 17](#_Toc152628078)

[**Figure S2.** Breakdown of the GDD harmonization process into actions-milestones and corresponding timelines. 18](#_Toc152628079)

[**Figure S3.** Outline of the GDD harmonization training presentation. 19](#_Toc152628080)

[**Figure S4.** Availability of nutrients across harmonized dietary datasets by country income level. 20](#_Toc152628081)

[**File S1.** Short Survey Questionnaire (SSQ). 21](#_Toc152628082)

[**File S2.** Survey Information (Metadata) form. 24](#_Toc152628083)

[**File S3.** List of abbreviations. 32](#_Toc152628084)

[**References** 33](#_Toc152628085)

**Table S1.** Materials developed to support the GDD harmonization process.^1^

| **Material** | **Material Description** | **Use** |
| --- | --- | --- |
| *Eligibility and prioritization of dietary surveys* | | |
| Eligibility criteria | The criteria developed to determine which surveys were eligible for the GDD harmonization. | Internal |
| Priority criteria | The criteria developed to determine which eligible surveys were to be prioritized by GDD. | Internal |
| *Dietary survey identification* | | |
| Survey list | Lists the 600 eligible surveys identified through survey identification; includes all survey characteristics required for survey prioritization; indicates the priority surveys; indicates which initiative (GDD or FAO/WHO GIFT) leads the harmonization. | Internal |
| *Survey contacting, screening, and inclusion* | | |
| GDD contact algorithm | Displays the GDD algorithm for contacting surveys under 4 different scenarios: 1) surveys contacted by GDD, 2) surveys introduced to GDD by EFSA, 3) surveys introduced to GDD by FAO, and 4) publicly available surveys. | Internal |
| GDD & FAO/WHO GIFT contact algorithm^2^ | Displays the contacting scheme between GDD and FAO to efficiently streamline contact of surveys of common interest without overburdening data owners. | Internal |
| Email templates | The email templates used to initiate the contacting process with data owners and for each different scenario included in the contact algorithm. | Internal |
| Contact status tracking sheet | Lists all surveys contacted by GDD along with their survey characteristics; includes and indicates surveys introduced to GDD by FAO & EFSA; tracks for each survey whether a response has been received, what was the response, exclusion (yes/no & why), completed materials received, and date of last contact. | Internal |
| GDD-FoodEx2 Overview | Provides a brief summary of the goal of the project and the collaboration with FAO and EFSA. The document was shared with data owners as part of our initial message to them. | External |
| [Short Survey Questionnaire (SSQ)](#_File_S1._Short) | Includes 9 questions, which determine final inclusion for each survey, provide key information on the current structure of the dataset, and indicate in what format the data owners are willing to share their data with GDD. The responses of the SSQ are used in combination with the financial incentive algorithm (see below) to decide the financial incentive available for this survey. | External |
| Data Sharing Agreement (DSA)^3^ | Lists the agreement terms pertaining to data sharing between Tufts and data owners. It is always signed by a duly authorized representative of the data owning institution. | External |
| Financial incentive algorithm | The algorithm the GDD developed to calculate the incentive to be offered to each survey based on the workload involved for its harmonization. | Internal |
| Financial incentive by survey | Lists all surveys financial incentive was offered to by GDD and includes the components for calculating the incentive, final incentive assigned, starting date of the payment process, and date of payment. | Internal |
| *Data retrieval and harmonization* | | |
| [Survey (Metadata) form](#_File_S2._Survey) | Collects detailed and standardized survey information, including survey representativeness, sampling design, sample, dietary assessment method, food classification system used, food composition table(s) used, and survey documentation, among others. The form was an adaptation of the FAO/WHO GIFT metadata form to avoid duplication of effort and ensure all key information are collected by both initiatives. | External |
| Microdata workbook | Contains detailed instructions for the data harmonization, as well as codebooks and templates for submitting variables related to participant sociodemographics dietary data. The workbook was an adaptation of EFSA’s relevant materials leveraging their long experience in collecting and harmonizing dietary microdata from different countries. | External |
| Harmonization process breakdown | Lists the sequence of actions and milestones to be completed for the GDD data harmonization. | External |
| Training presentation^4^ | Provides detailed information, instructions, and key notes for the GDD data harmonization. It is divided in 3 sections: 1) general process of the data harmonization, including a timeline with specific milestones, 2) instructions for filling all forms and templates, and 3) FoodEx2 training, including key rules and examples. | External |
| FoodEx2 mapping exercise^4^ | Lists 32 foods to be mapped with FoodEx2 by data owners before the training webcall. | External |
| FoodEx2 browser installation guide^4^ | Contains detailed instructions for installing the FoodEx2 browser and catalogue. | External |
| Training call presentation template^4^ | The template for the presentation used during the training webcall delivered to data owners by the GDD team. It included a summary of the theory presented in the detailed training presentation along with a survey-specific timeline for the completion of the harmonization. | External |
| *Quality control* | | |
| Data cleaning steps | Contains the step-by-step description of the data processing, checking, cleaning, and management procedures that the GDD team followed as part of the data harmonization process. | Internal |
| *Data Dissemination* | | |
| Acknowledgement templates | Lists all surveys to be harmonized and the acknowledgments that should be used whenever the harmonized data is used. The survey citation was provided by the data owners. | Internal |

^1^ All material were developed by the GDD, unless otherwise specified; FAO reviewed these and provided high-level input. Despite leveraging each other’s experience and expertise and trying to harmonize the methods followed to the extent possible, GDD and FAO used their own processes and materials for harmonizing dietary microdata. In particular, the two initiatives have their own eligibility and priority criteria, email templates, data sharing agreement, metadata form, microdata workbook, training materials, data cleaning steps and acknowledgement templates, while there are materials that were only part of the GDD harmonization process, including the GDD contact algorithm, the contact status tracking sheet, GDD-FoodEx2 Overview, Short Survey Questionnaire, financial incentive algorithm, financial incentive by country, harmonization process breakdown, and training presentation.

^2^ Developed by FAO with input from the GDD; this material did not differ across the two initiatives.

^3^ The Data Sharing Agreement (DSA) was an update of the previous GDD DSA version.

^4^ Reviewed by the European Food Safety Authority (EFSA).

**Table S2.** Criteria for determining survey eligibility for the GDD dietary data harmonization.

| **Search limits** | |
| --- | --- |
| Language | No limits |
| Publication date | 1980 - present |
| **Eligibility (inclusion) criteria^1^** | |
| Population | General population as well as specific population subgroups (e.g., children, pregnant/lactating women) |
| Population age | All ages |
| Selection bias | No evidence of strong selection bias. The survey sample should not be limited to a particular subgroup whose diet would significantly differ from the general population, e.g. only diabetics, only overweight/obese individuals, only specific ethnic minorities or immigrants |
| Representativeness | Survey nationally, sub-nationally, or community representative |
| Sample size | Survey must have a sample size of greater than 100 |
| Foods/beverages covered | Capture whole diet to the extent possible |
| Diet assessment method | 24-hour recall or food record/diary |
| Number of reported unique food items | ≥50 |
| Level of description detail of reported food items | EFSA’s minimum level of detail for FoodEx2 mapping are met. Foods should not be reported as generic categories (e.g., fruit, cereal, meat) but rather, in as specific terms as possible (e.g., apple, wheat grain, bovine meat) |
| Data sharing agreement | Agreement to make the harmonized microdata publicly available through the GDD platform |

EFSA, European Food Safety Authority; GDD, Global Dietary Database.

^1^ All criteria should be met to characterize a survey as eligible for dietary data harmonization.

**Table S3.** Criteria for prioritizing eligible surveys for the GDD dietary data harmonization.

| **Top priority criteria** | **High priority given to:** |
| --- | --- |
| Survey repetition | Rolling programs^1^ or expected to be |
| Representativeness | Nationally and subnationally representative surveys (local surveys are of low priority) |
| Countries of interest to funding organization | Surveys in Bangladesh, Burkina Faso, Ethiopia, India, and Nigeria |
| **Other factors to consider when selecting a survey for harmonization** | **Preferred surveys** |
| Country size | From countries of greater size |
| Gender | Including both males and females; though surveys can be specific to e.g., only women of reproductive age |
| Age | Including all ages (or wider age range); though surveys can be specific to a certain age subgroup (e.g., children) |
| Quality of sampling methodology | Of reasonable sampling design quality |
| Coverage | Covering both urban and rural areas |
| Data collection period | More recent surveys |
| Quality of diet assessment | Of reasonable diet assessment quality |
| Number of unique food items | With greater number of food items reported |
| Country income level | Representing roughly equally all country income levels (LIC, LMIC, UMIC, HIC)^2^ |
| Language^3^ | With data reported in English or Spanish |
| FoodEx2 availability | With FoodEx2 mapping already in place |
| Responsiveness of data owners | With highly responsive data owners |

^1^ Rolling programs refer either to surveys repeated every (few) year(s) (e.g., NHANES) or to cohort studies.

^2^ Country-income level is determined based on the World Bank classification of countries into low-income (LIC), lower-middle income (LMIC), upper-middle income (UMIC), and high-income (HIC) countries.^1^

^3^ Only applicable to publicly available datasets and given that the data harmonization would be performed by the GDD team without the involvement of data owners.

**Table S4.** Variable definitions and values for the survey participant data (Participant Codebook).

| **Variable name** | **Variable definition** | **Status** | **Type of variable** | **Variable label** | **Variable values** |
| --- | --- | --- | --- | --- | --- |
| id | Unique numeric identifier of the participant | Compulsory | Numeric | Participant id |  |
| hh_id | Unique numeric identifier for the household | Optional | Numeric | Household id |  |
| country | Country of the survey | Compulsory | Text | Country |  |
| sec_area | Region of the country - Secondary geographical/administrative area | Optional | Text | Region |  |
| tert_area | Sub-region of the country - Tertiary geographical/administrative area | Optional | Text | Subregion |  |
| round | Round number. If the survey was not carried out over different seasons or clear separated time spans, the round number will always be 1 | Compulsory | Numeric, 3 digits or less | Survey round/ collection period |  |
| smpl_weight | The sampling weight estimated for every participant, to correct for potential under- or over-representation of some groups in the sample and reinstate the original importance of each group within the population | Compulsory | Numeric, non-zero, all available decimal digits | Sampling weight | **Important:** if >1 sampling weights are available for each participant (e.g., for interview, dietary data collection, clinical examination), please, create separate variables, further indicating what each of the sampling weights represent and how they should be used. |
| sex | Sex of the participant | Compulsory | Dichotomous | Participant sex | 1 - Male 2 - Female |
| age | Age of the participant in years | Compulsory | Numeric, all available decimal digits | Participant age (years) | Age in years. For all ages ≥ 2 years, enter the year as a whole number. For children <2 years, enter the age as a decimal (1 month=0.08 years, 2 months=0.17, 3 months=0.25, 4 months=0.33, 5 months=0.42, 6 months=0.5, 7 months=0.58, 8 months=0.67, 9 months=0.75, 10 months=0.83, 11 months=0.92, 12 months=1, 13 months=1.08 etc.). |
| edc_level | Education level of the participant | Compulsory | Ordinal | Participant education level | 1 - Primary (0 to ≤6 years of formal education or ISCED levels =<1)  2 - Secondary (more than primary school through high school graduate; e.g., >6 to ≤12 years of formal education or ISCED levels 2-3) 3 - Tertiary (any education beyond high school, e.g., >12 years of formal education or ISCED levels >=4) Note: for children/adolescents, the level of education refers to that of the head of household |
| edc | Highest degree (or class) completed by the participant | Optional | Text | Participant education (highest degree completed) |  |
| residence | Type of area in which the participant is living | Compulsory | Dichotomous | Urban/Rural residence | 1 - Rural 2 - Urban |
| preg_lact | Pregnant or lactating status of female participants | Compulsory | Nominal | Pregnant/Lactating status | 0 - Not pregnant/lactating 1 - Pregnant 2 - Lactating 3 - Pregnant & lactating |
| breastfeeding | Specify whether the child is breastfeeding, for children up to 5 years old | Compulsory | Nominal | Breastfeeding children | 0 - No 1 - Partially breastfeeding 2 - Exclusively breastfeeding 3 - Breastfeeding (unspecified if partially or exclusively) |
| hh_head | Household member reported as being the head of the household | Optional | Numeric | Household head | *Indicate the id of the participant that is considered the household head* |
| wgt | Body weight of the participant in kg. If multiple measurements have been performed provide the measurement you consider more accurate for the participants | Compulsory | Numeric, all decimal digits available | Participant body weight (kg) |  |
| wgt_method | Method used to measure body weight | Compulsory | Dichotomous | Method of body weight measurement | 1 - Measured 2 - Self-reported |
| hgt | Height of the participant in cm. If multiple measurements have been performed provide the measurement you consider more accurate for the participants | Compulsory | Numeric, all decimal digits available | Participant height (cm) |  |
| hgt_method | Method used to measure height | Compulsory | Dichotomous | Method for height measurement | 1 - Measured 2 - Self-reported |
| bmi_adults | Body mass index (BMI) defined as weight/(height)² for participants >=20 years old | Compulsory | Numeric, all decimal digits available | BMI adult participants (kg/m²) |  |
| bmi_children | Body Mass Index (BMI) age- and sex- specific percentile for participants <20 years old | Compulsory | Numeric | BMI children (percentile) |  |
| bmi_cat | Participant classification based on BMI for adults (≥20 years old) and on BMI age- and sex- specific percentile for children/adolescents (<20 years old) | Compulsory | Nominal | BMI classification | 1 - Underweight (Adults ≥20y: BMI <18.5 kg/m²; Children <20y: BMI <5th age- and sex- specific percentile) 2 - Normal (Adults ≥20y: BMI 18.5-24.9 kg/m²; Children <20y: BMI 5th to <85th age- and sex- specific percentile) 3 - Overweight (Adults ≥20y: BMI 25-29.9 kg/m²; Children <20y: BMI 85th to <95th age- and sex- specific percentile) 4 - Obese (Adults ≥20y: BMI ≥30 kg/m²; Children <20y: BMI ≥95th age- and sex- specific percentile) |
| misrep | Participant identified as under- or over-reporter of energy | Optional | Nominal | Energy misreporting | 1 - Under-reporter 2 - Normal 3 - Over-reporter |
| pa | Physical activity level of the participant in Metabolic Equivalents of Task (METs) minutes per week | Compulsory | Numeric, 5 digits or less | MET-min per week |  |
| pa_cat | Physical activity level of the participant in categories | Compulsory | Ordinal | Physical activity classification | 1 - Low (<600 MET-min/week) 2 - Moderate (600-2,999 MET-min/week) 3 - High (>=3,000 MET-min/week) |
| smok | Smoking status of the participant | Compulsory | Nominal | Smoking status | 1 - Smoker 2 - Former smoker 3 - Never smoker 4 - Non-smoker - unspecified if former or ever |
| special_diet | Particular dietary pattern that the participant followed at the time of the dietary data collection | Optional | Nominal | Special diet | 0 - No special diet 1 - Vegetarian diet 2 - Vegan diet 3 - Weight loss diet 4 - Weight gain diet 5 - Other |
| special_diet_other | Other particular dietary pattern that the participant followed at the time of the dietary data collection | Optional | Text | Other special diet |  |
| clin_diet | Particular dietary pattern that the participant followed at the time of the dietary data collection due to a health condition | Optional | Nominal | Special condition diet | 0 - No clinical diet 1 - Low fat or cholesterol diet 2 - Low salt or sodium diet 3 - Diabetic diet 4 - Renal or kidney diet 5 - Gluten-free or celiac diet 6 - Other |
| clin_diet_other | Other particular dietary pattern that the participant followed at the time of the dietary data collection due to a health condition | Optional | Text | Other special condition diet |  |
| note | Any relevant comments for the participant that may have implications for the analysis | Optional | Text | Notes |  |

**Table S5.** Variable definitions and values for the dietary data (Diet Codebook).

| **Variable name** | **Variable definition** | **Unit** | **Status** | **Type of variable** | **Variable label** | **Variable values** |
| --- | --- | --- | --- | --- | --- | --- |
| id | Unique numeric identifier of the participant |  | Compulsory | Numeric | Participant id |  |
| sex | Sex of the participant |  | Compulsory | Dichotomous | Participant sex | 1 - Male 2 - Female |
| recall_n | The ordinal number of recall/ record collected for the participant |  | Compulsory | Numeric, 1 digit | Recall number | **Important:** if, for example, 2 recalls have been collected for the participant, then the value 1 is entered for dietary data of the first recall and 2 is entered for dietary data of the second recall) |
| recall_d | Recall/Record collection date - Day |  | Compulsory | Numeric, 2 digits or less | Recall day | From 1 to 31 |
| recall_m | Recall/Record collection date - Month |  | Compulsory | Numeric, 2 digits or less | Recall month | From 1 to 12 |
| recall_y | Recall/Record collection date - Year |  | Compulsory | Numeric, 4 digits | Recall year |  |
| weekday | Day of the week that the recall/record was collected |  | Compulsory | Nominal | Day of the week | 1 - Monday 2 - Tuesday 3 - Wednesday 4 - Thursday 5 - Friday 6 - Saturday 7 - Sunday |
| respondent | Respondent of the dietary assessment method |  | Compulsory | Nominal | Respondent | 1 - Self 2 - Person responsible for cooking 3 - Parent/Guardian 4 - Other relative 5 - Nanny/Nurse/Friend/Neighbor 6 - Proxy (unspecified) |
| us_intake | Self-reported (i.e., as reported by the participant) specification of whether the dietary intake reported corresponds to the usual intake of the participant (e.g., the participant may had an outside-the-usual dietary intake the day for which dietary data were collected, because of a special occasion/event (e.g., sickness, party), holiday, long working hours etc.) |  | Optional | Nominal | Usual intake | 0 - Usual intake 1 - Higher intake than usual 2 - Lower intake than usual 3 - Not usual intake, unspecified if higher or lower |
| consum_h | Time of consumption (hours) |  | Optional | Numeric, 2 digits | Time of consumption (hours) | From 00 to 23 |
| consum_min | Time of consumption (minutes) |  | Optional | Numeric, 2 digits | Time of consumption (minutes) | From 00 to 59 |
| meal_type | Type of the consumed meal |  | Optional | Nominal | Meal type | 1 - Before breakfast  2 - Breakfast 3 - Snack between breakfast and lunch 4 - Lunch 5 - Snack between lunch and dinner 6 - Dinner 7 - Snack after dinner |
| meal_place | Place the meal was consumed |  | Optional | Dichotomous | Place of consumption | 1 - Home 2 - Out of home |
| eat_seq | Ordinal number of the eating occasion within the meal. Each simple food or mixed dish/recipe determines an eating occasion |  | Optional | Numeric | Number of eating occasion |  |
| food_type | Type of food item consumed (i.e., simple food or mixed dish/recipe) |  | Compulsory | Nominal | Type of consumed food | 1 - Simple food item (1-ingredient food items) 2 - Reported mixed dish/recipe (>1-ingredient food items; the ingredients and their amounts are reported by the respondent)  3 - Standard mixed dish/recipe (>1-ingredient food items; the ingredients and their amounts are derived from a source that describes recipes commonly consumed by the population (e.g., food composition table)) 4 - Non-disaggregated mixed dish/recipe (>1-ingredient food items; disaggregation is not possible (e.g., mass-produced packaged sweet or savoury snacks, such as biscuits and cakes) or not available) |
| rcp_code | Unique identifier of the mixed dish/recipe if applicable (code to be repeated for each ingredient belonging to the recipe) as appears originally in the dataset |  | Compulsory | Text | Recipe unique identifier |  |
| rcp_descr | Description of the mixed dish/recipe |  | Compulsory | Text | Recipe description (local language) |  |
| rcp_descr_eng | Description of the mixed dish/recipe in English. |  | Compulsory | Text | Recipe description (English) |  |
| foodex2_rcp_code | FoodEx2 code for the recipe (with facet if necessary) |  | Optional | Text | Recipe FoodEx2 code |  |
| foodex2_rcp_descr | FoodEx2 description of the recipe |  | Optional | Text | Recipe FoodEx2 description |  |
| rcp_amount | Amount consumed of the total mixed dish/recipe | g | Compulsory | Numeric, 6 digits or less (including 2 decimal digits or less) | Amount recipe consumed (g) |  |
| ingr_code | Unique identifier of the simple food or mixed dish/recipe ingredient as appears originally in the dataset |  | Compulsory | Numeric | Simple food item unique identifier |  |
| ingr_descr | Description of the simple food or mixed dish/recipe ingredient |  | Compulsory | Text | Simple food item description (local language) |  |
| ingr_descr_eng | Description of the simple food or mixed dish/recipe ingredient in English |  | Compulsory | Text | Simple food item description (English) |  |
| foodex2_ingr_code | FoodEx2 code of the simple food or mixed dish/recipe ingredient (with facet if necessary) |  | Compulsory | Text | FoodEx2 simple food/ingredient code |  |
| foodex2_ingr_descr | FoodEx2 description of the simple food or mixed dish/recipe ingredient |  | Compulsory | Text | FoodEx2 simple food/ingredient description |  |
| ingr_amount_unproc | Edible (e.g., without bones, peels) amount of the simple food (or the mixed dish/recipe ingredient) consumed before processing | g | Optional | Numeric, all decimal digits available | Amount (edible) consumed before processing (g) |  |
| ingr_amount_proc | Edible amount of the simple food (or the ingredient of a mixed dish/recipe) consumed after processing/cooking | g | Compulsory | Numeric, all decimal digits available | Amount (edible) consumed after processing (g) |  |
| energy | Energy content | kcal | Compulsory | Numeric, all decimal digits available | Energy intake (kcal) |  |
| totalpro | Total protein content | g | Compulsory | Numeric, all decimal digits available | Protein intake (g) |  |
| animalpro | Meat (red meat, poultry, fish, egg) protein content | g | Compulsory | Numeric, all decimal digits available | Animal protein intake (g) |  |
| dairypro | Dairy protein content | g | Compulsory | Numeric, all decimal digits available | Dairy protein intake (g) |  |
| plantpro | Plant protein content | g | Compulsory | Numeric, all decimal digits available | Vegetal protein intake (g) |  |
| carb | Total carbohydrate content | g | Compulsory | Numeric, all decimal digits available | Carbohydrate intake (g) |  |
| adsugar | Total content of sugars added during the preparation or processing of foods and beverages. Examples include the sugars added in sugar-sweetened beverages, desserts, candy, breakfast cereals, and sweetened milk. This definition excludes non-caloric sweeteners and sugars that naturally occur in foods, such as those in fruits, milk or milk-products | g | Compulsory | Numeric, all decimal digits available | Added sugar intake (g) |  |
| fiber | Total dietary fiber intake, defined as the carbohydrate polymers which are not hydrolyzed by the endogenous enzymes in the small intestine of human beings. Dietary fiber should optimally be quantified using the AOAC method of analysis | g | Compulsory | Numeric, all decimal digits available | Fiber intake (g) |  |
| totalfat | Total fat content | g | Compulsory | Numeric, all decimal digits available | Total fat intake (g) |  |
| sfa | Total saturated fat content | g | Compulsory | Numeric, all decimal digits available | Saturated fat intake (g) |  |
| mufa | Total monounsaturated fat content | g | Compulsory | Numeric, all decimal digits available | Monounsaturated fat intake (g) |  |
| pufa | Total poly-unsaturated fat content | g | Compulsory | Numeric, all decimal digits available | Polyunsaturated fat intake (g) |  |
| water | Total water content | g | Compulsory | Numeric, all decimal digits available | Water intake (g) |  |
| n6 | Total omega-6 fatty acid content | g | Compulsory | Numeric, all decimal digits available | Omega-6 fatty acid intake (g) |  |
| seafood_n3 | Total dietary EPA+DHA (eicosapentaenoic acid + docosahexaenoic acid) content | g | Compulsory | Numeric, all decimal digits available | Seafood omega-3 fatty acid intake (g) |  |
| plant_n3 | Total dietary ALA (alpha-linolenic acid) content | g | Compulsory | Numeric, all decimal digits available | Plant omega-3 (n-3) fattty acid intake (g) |  |
| tfa | Total trans fatty acid content | g | Compulsory | Numeric, all decimal digits available | Trans fatty acid intake (g) |  |
| chol | Cholesterol content | mg | Compulsory | Numeric, all decimal digits available | Cholesterol intake (mg) |  |
| ca | Calcium content | mg | Compulsory | Numeric, all decimal digits available | Calcium intake (mg) |  |
| fe | Total intake of heme and non-heme iron content | mg | Compulsory | Numeric, all decimal digits available | Iron intake (mg) |  |
| na | Sodium content | mg | Compulsory | Numeric, all decimal digits available | Sodium intake (mg) |  |
| k | Potassium content | mg | Compulsory | Numeric, all decimal digits available | Potassium intake (mg) |  |
| mg | Magnesium content | mg | Compulsory | Numeric, all decimal digits available | Magnesium intake (mg) |  |
| zn | Zinc content | mg | Compulsory | Numeric, all decimal digits available | Zinc intake (mg) |  |
| iod | Iodine content | mg | Compulsory | Numeric, all decimal digits available | Iodine intake (mg) |  |
| se | Selenium content | mg | Compulsory | Numeric, all decimal digits available | Selenium intake (mg) |  |
| ph | Phosphorus content | mg | Compulsory | Numeric, all decimal digits available | Phosphorus intake (mg) |  |
| cu | Copper content | mg | Compulsory | Numeric, all decimal digits available | Copper intake (mg) |  |
| vitb1 | Thiamine content | mg | Compulsory | Numeric, all decimal digits available | Thiamine intake (mg) |  |
| vitb2 | Riboflavin content | mg | Compulsory | Numeric, all decimal digits available | Riboflavin intake (mg) |  |
| vitb3 | Niacin content | mg | Compulsory | Numeric, all decimal digits available | Niacin intake (mg) |  |
| vitb6 | Total vitamin B6 content (including 2-methyl, 3-hydroxy, 5-hydroxymetrhyl pyridine derivatives that exhibit the nutritional activity of pyridoxine) | mg | Compulsory | Numeric, all decimal digits available | Vitamin B6 intake (mg) |  |
| fol | Total Vitamin B9 (including folate and folic acid) content | μg DFE | Compulsory | Numeric, all decimal digits available | Folate intake (µg DFE) |  |
| vitb12 | Total cobalamin content (including cyanocobalamin, hydroxocobalamin, aquocobalamin, sulfitocobalamin, etc.) | μg | Compulsory | Numeric, all decimal digits available | Vitamin B12 intake (µg) |  |
| vitc | Vitamin C content | mg | Compulsory | Numeric, all decimal digits available | Vitamin C intake (mg) |  |
| vita | Total Vitamin A (including retinol, retinal, retinoic acid, and retinyl esters) and provitamin A carotenoid content | μg RAE | Compulsory | Numeric, all decimal digits available | Vitamin A intake (µg RAE) |  |
| bcarot | B-carotene content | μg | Compulsory | Numeric, all decimal digits available | B-carotene intake (μg) |  |
| vitd | Total vitamin D content (including vitamin D2, vitamin D3, and vitamin D provitamins and previtamins) | μg | Compulsory | Numeric, all decimal digits available | Vitamin D intake (µg) |  |
| vite | Total vitamin E (tocopherols and tocotrienols) content | mg | Compulsory | Numeric, all decimal digits available | Vitamin E (alpha-tocopherol equivalents) or [alpha-tocopherol] intake (mg) |  |
| vitk | Total vitamin K content | μg | Compulsory | Numeric, all decimal digits available | Vitamin K intake (µg) |  |
| note | Add any relevant comments for the participant that may have implications for the analysis (e.g., non-plausible intakes for total grains but kept in the dataset for xxx reasons) |  | Optional | Text | Notes |  |

**Table S6.** Top-level food groups of the FoodEx2 classification system.^1^

| **N** | **Food group** |
| --- | --- |
| 1 | Grains and grain-based products |
| 2 | Vegetables and vegetable products |
| 3 | Starchy roots or tubers and products thereof, sugar plants |
| 4 | Legumes, nuts, oilseeds and spices |
| 5 | Fruit and fruit products |
| 6 | Meat and meat products |
| 7 | Fish, seafood, amphibians, reptiles and invertebrates |
| 8 | Milk and dairy products |
| 9 | Eggs and egg products |
| 10 | Sugar and similar, confectionery and water-based sweet desserts |
| 11 | Animal and vegetable fats and oils and primary derivatives thereof |
| 12 | Fruit and vegetable juices and nectars (including concentrates) |
| 13 | Water and water-based beverages |
| 14 | Alcoholic beverages |
| 15 | Coffee, cocoa, tea and infusions |
| 16 | Food products for young population |
| 17 | Products for non-standard diets, food imitates and food supplements |
| 18 | Composite dishes |
| 19 | Seasoning, sauces and condiments |
| 20 | Major isolated ingredients, additives, flavours, baking and processing aids |
| 21 | Other ingredients |

^1^ Adapted from EFSA’s technical report on FoodEx2.^2^ The reported top-level food groups refer to the exposure hierarchy of FoodEx2 which is specific to food consumption.

**Table S7.** The facets of the FoodEx2 description system.^1^

| **Facet code** | **Facet** | **Facet description** |
| --- | --- | --- |
| F01 | Source | Defines the origin food of raw commodities. |
| F02 | Part-nature | Defines the generic types of food, answering the question ‘what type of food is this?’ |
| F03 | Physical state | Distinguishes between two forms of the same food (e.g., liquid vs solid, powder vs bar). |
| F04 | Ingredient | Defines the ingredients of composite foods. |
| F06 | Surrounding medium | Specifies the medium in which the food is preserved/packed. Mainly used for canned or jarred foods. |
| F07 | Fat-content | Specifies the amount of fat present in the food as a percentage. |
| F08 | Sweetening agent | Specifies the sweetener of foods. |
| F09 | Fortification agent | Specifies the substance with which the food is fortified. |
| F10 | Qualitative info | Defines several characteristics of the food. There are different sections within this facet that address separate aspects (e.g., reduced fat, without added sugar, low in sodium, integral/not refined, lactose-free) |
| F11 | Alcohol content | Specifies the amount of alcohol present in the beverage as a percentage. Only relevant for the category of alcoholic beverages, when the alcohol content is not obvious. |
| F12 | Dough mass | Defines the type of dough used in the preparation of the food. Only relevant for bakery products when not obvious. |
| F17 | Extent of cooking | Defines characteristics derived from the length or type of cooking. Important for products such as meat, vegetables, and eggs. |
| F18 | Packaging format | Specifies the type of food packaging (e.g., bottle, box). |
| F19 | Packaging material | Specifies the material of the food packaging (e.g., glass, paper). |
| F20 | Part consumed-analysed | Specifies further the part of the food consumed (e.g., with peel, with skin, without visible fat). |
| F21 | Production method | Specifies the method used for the preparation of the food. Important in cases such as organic farming or aquaculture. |
| F22 | Preparation-production place | Specifies where the food was produced (e.g., food industry restaurant). |
| F23 | Target consumer | Indicates by whom the food was designed to be consumed (e.g. for infants, for diabetics, for weight reduction). Only used when information is not obvious or when the information makes the difference. |
| F24 | Intended use | Specifies how is the food intended to be consumed (e.g., ready/not ready to eat, intended to be eaten raw/cooked). Only used with the microbiological domain. |
| F25 | Risky ingredient | Indicates when the food was prepared using ingredients considered of risk (e.g., containing raw cream). Only used for the microbiological domain. |
| F26 | Generic term | Indicates when the food is unspecified or is not included in the browser. |
| F27 | Source commodities | Defines the origin of derivatives. |
| F28 | Process | Specifies processes used for the production/preparation of the food (e.g., pasteurization, frying, boiling, mincing, chocolate coating). |
| F29 | Purpose of raising | Specifies the reason why the animal was grown. Not relevant for food consumption. |
| F30 | Reproductive level | Specifies the level of breeding. Not relevant for food consumption. |
| F31 | Animal age class | Specifies the age of the animal. Not relevant for food consumption. |
| F32 | Gender | Specifies the gender of the animal. Not relevant for food consumption. |
| F33 | Legislative classes | Specifies legislative class to which the food belongs. Important for some legislation-oriented data collection. |

^1^ Adapted from EFSA’s technical report on FoodEx2.^2^

# **Figure S1.** The Global Dietary Database contact process flowchart.


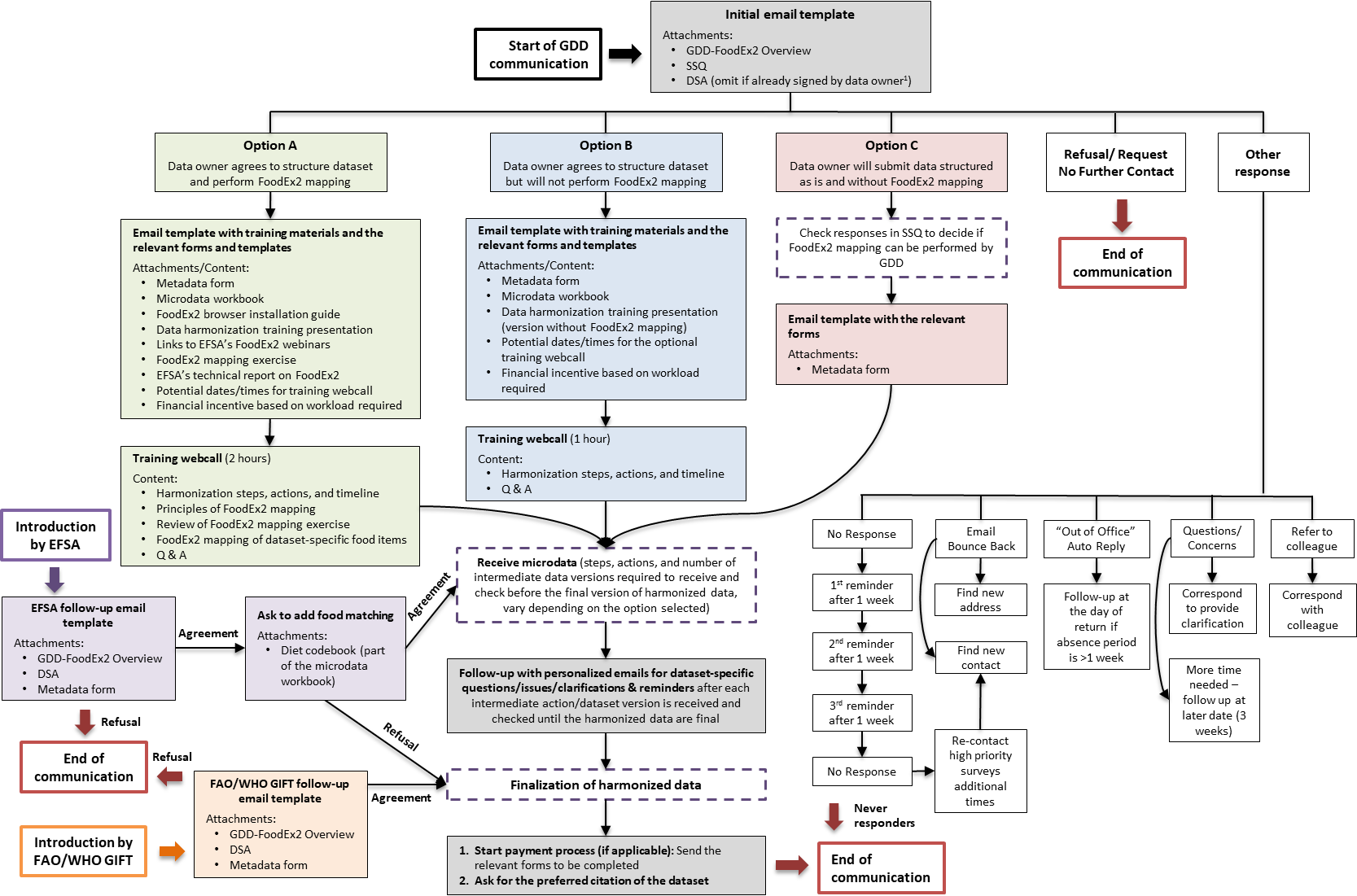


# **Figure S2.** Breakdown of the GDD harmonization process into actions-milestones and corresponding timelines.


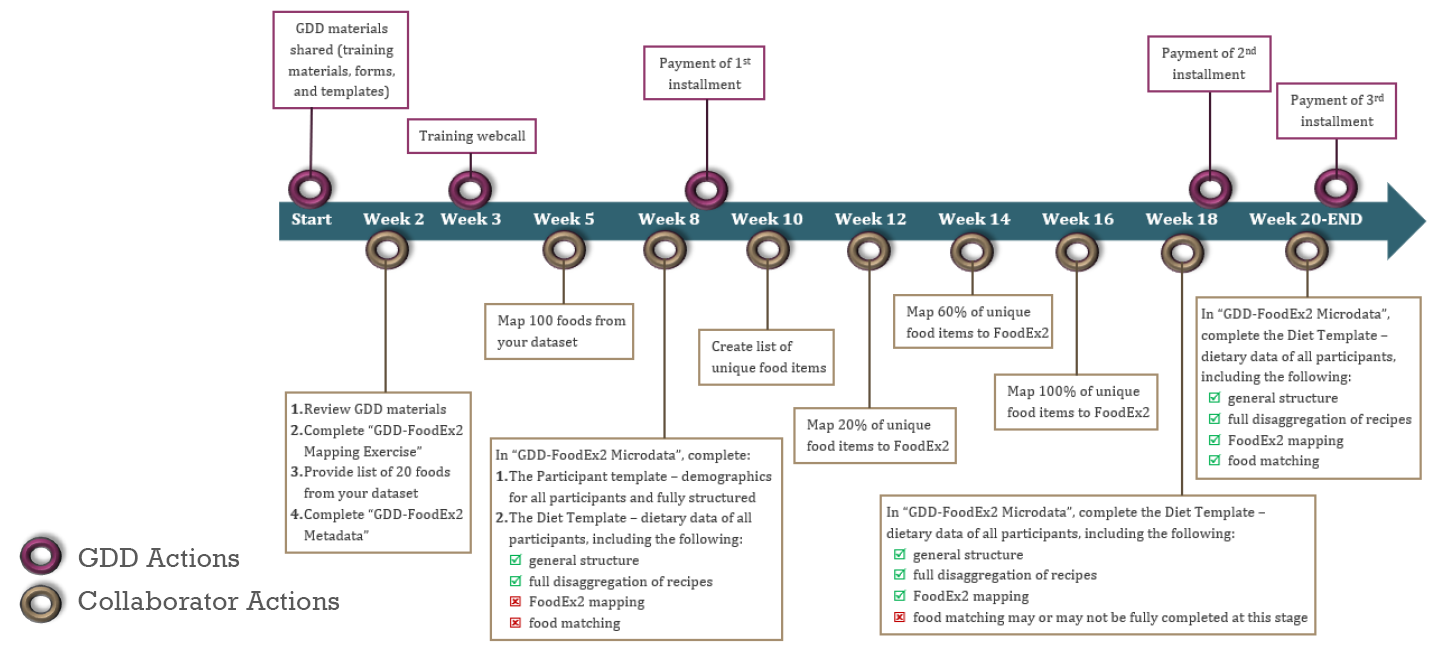


# **Figure S3.** Outline of the GDD harmonization training presentation.


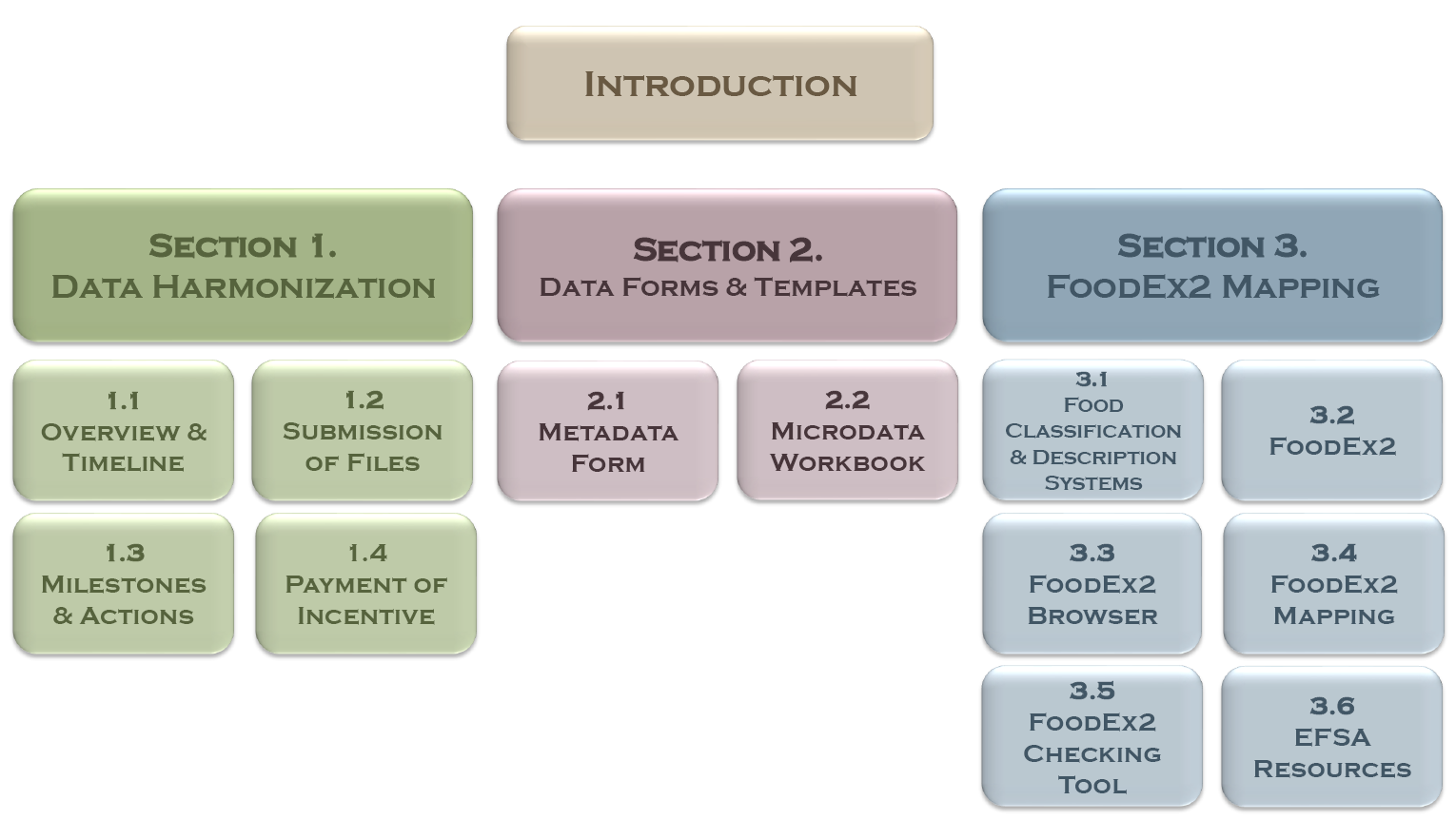


# **Figure S4.** Availability of nutrients across harmonized dietary datasets by country income level.


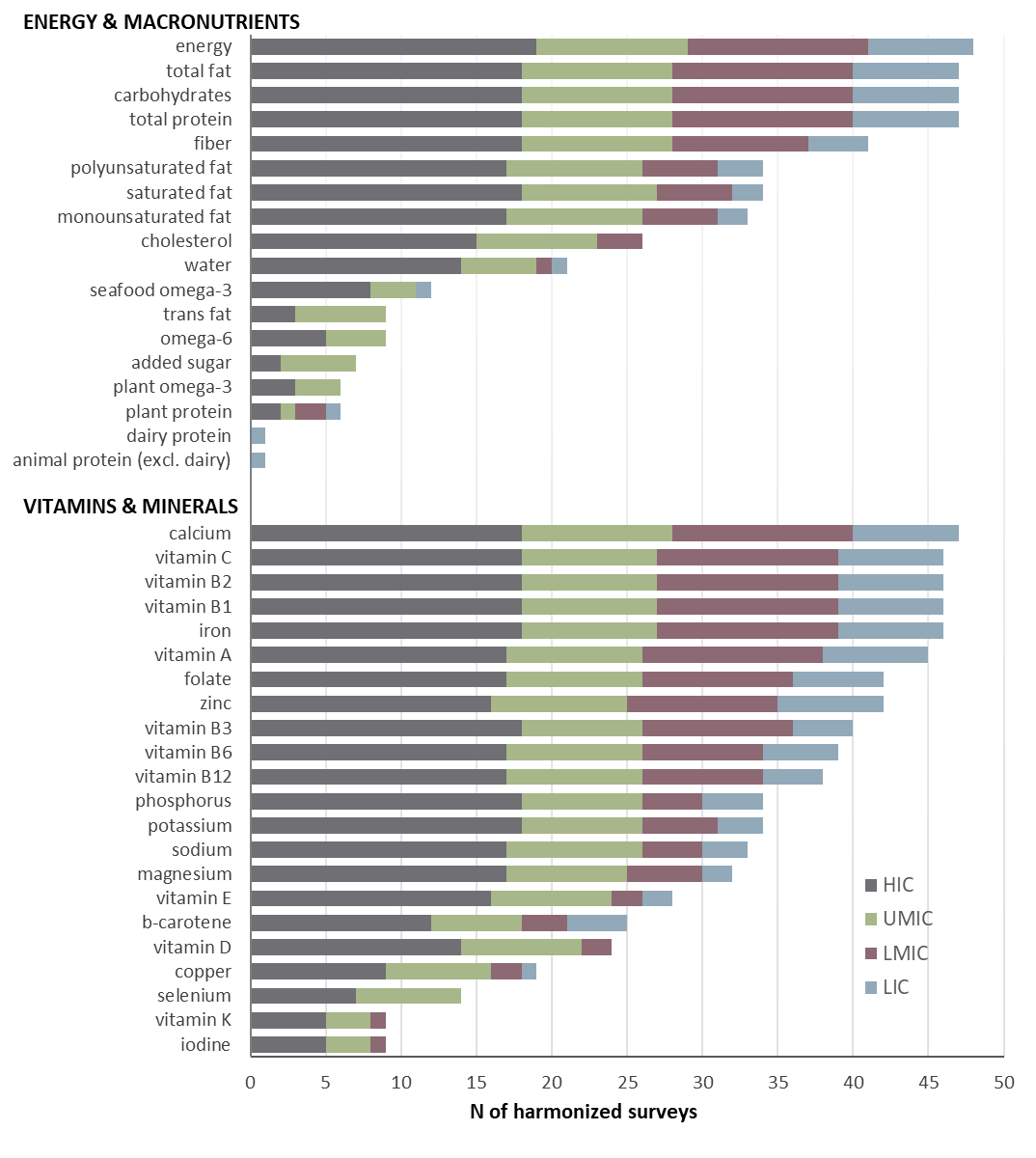


Only surveys whose harmonization has been completed and they report nutrient intakes are presented (N=48 of 55). The figure presents the 40 nutrients requested by GDD; datasets may contain additional nutrients (e.g., total sucrose, retinol) that are not listed here. Country-income level is determined based on the World Bank classification of countries into low-income (LIC), lower-middle income (LMIC), upper-middle income (UMIC), and high-income (HIC) countries.^1^ The definitions and units of measurement for nutrients are available in Table S5.

# **File S1.** Short Survey Questionnaire (SSQ).


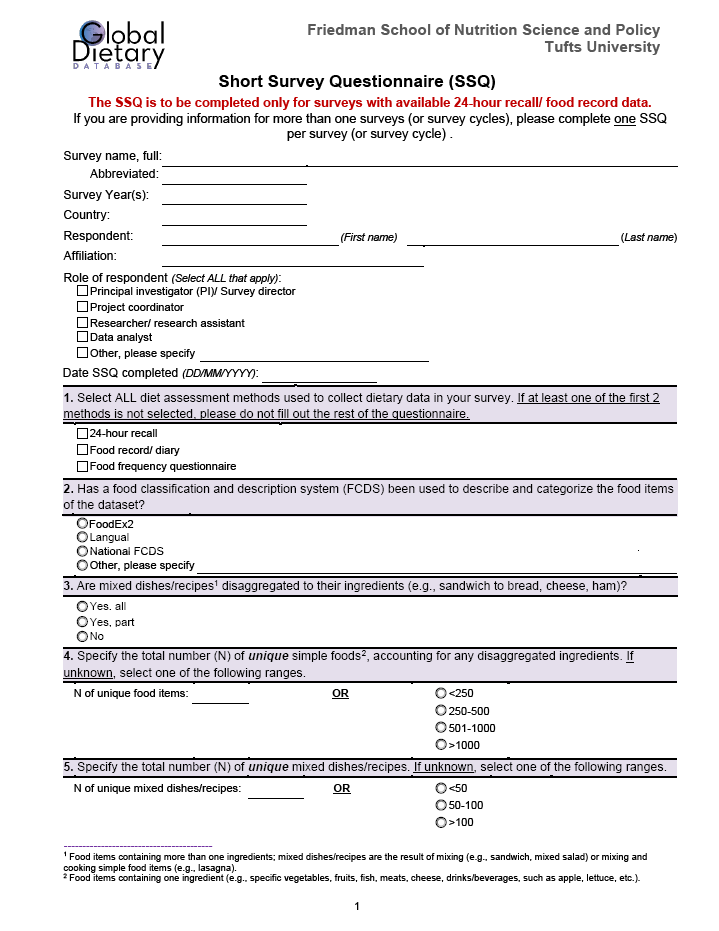


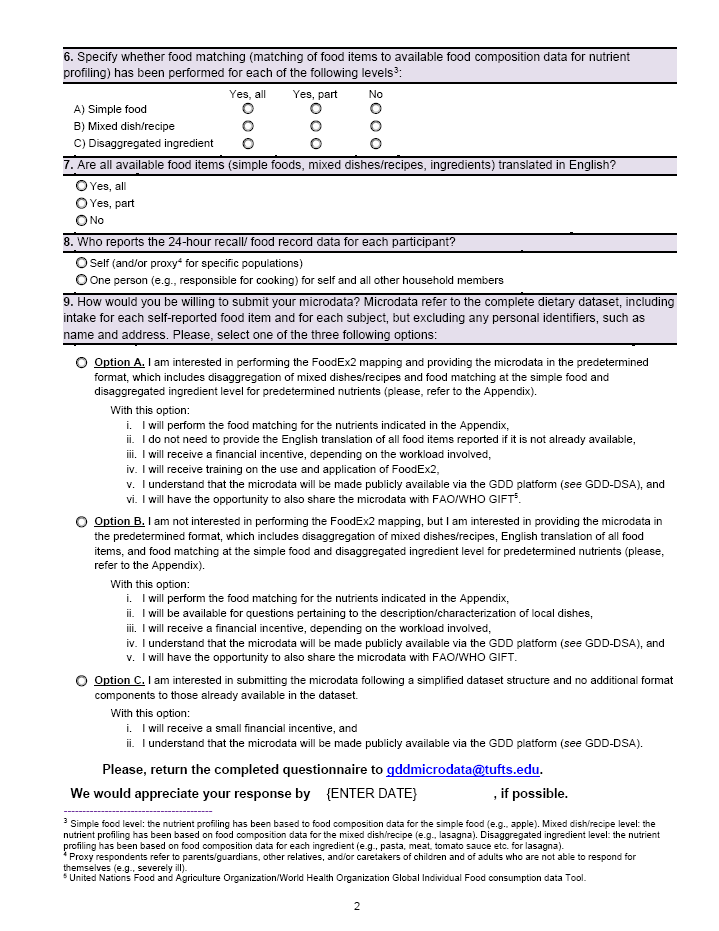


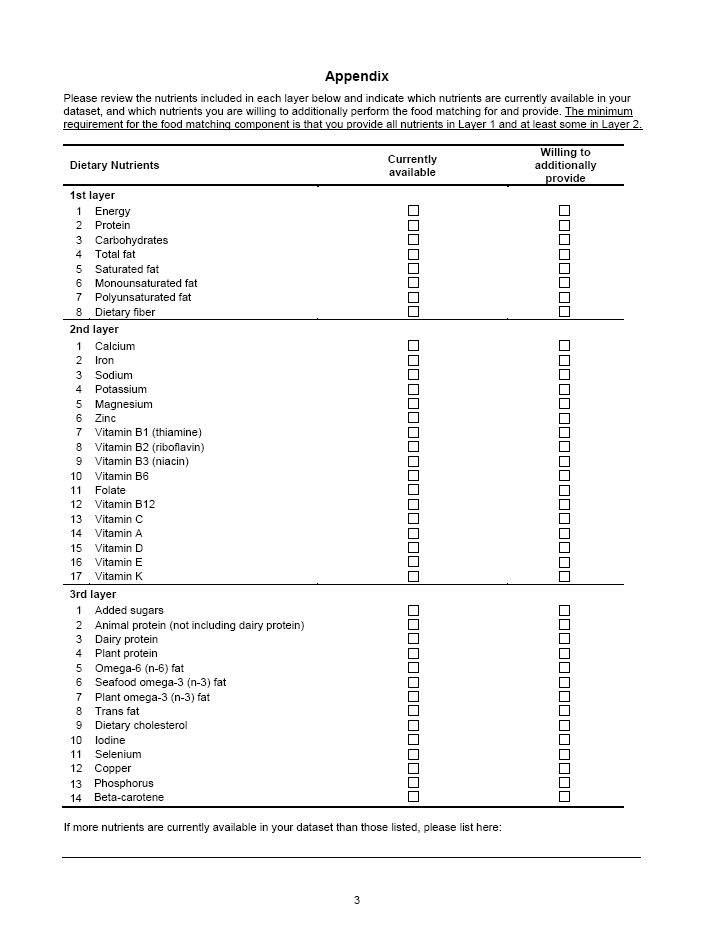


# **File S2.** Survey Information (Metadata) form.


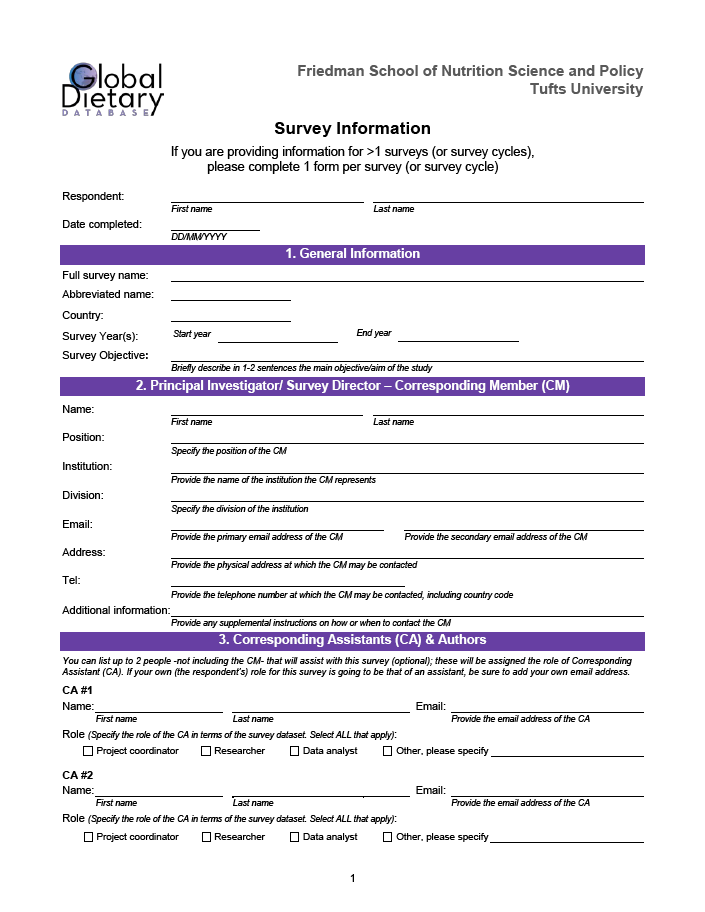


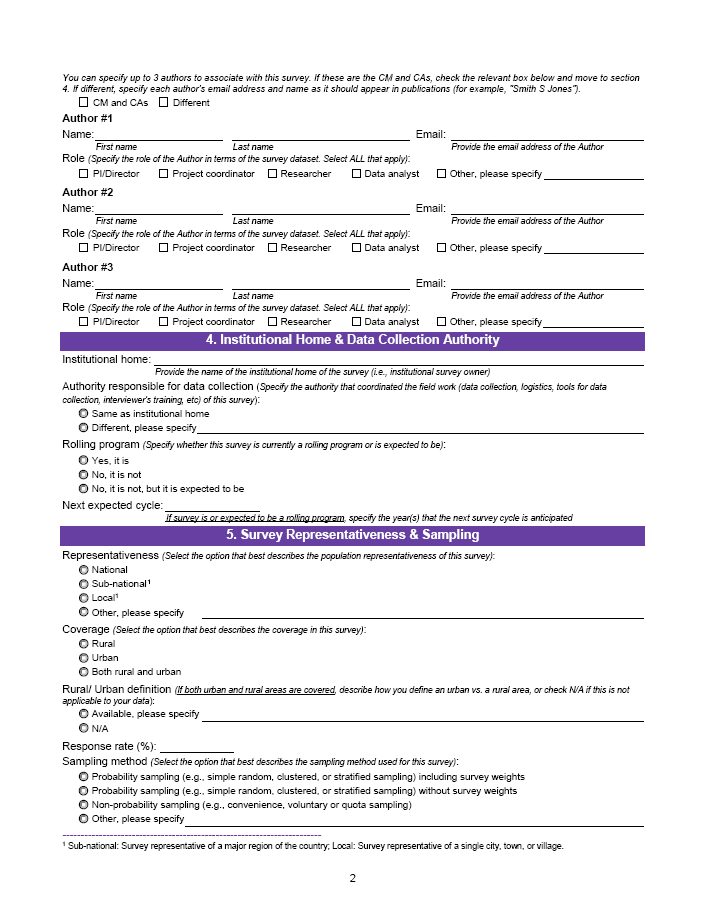


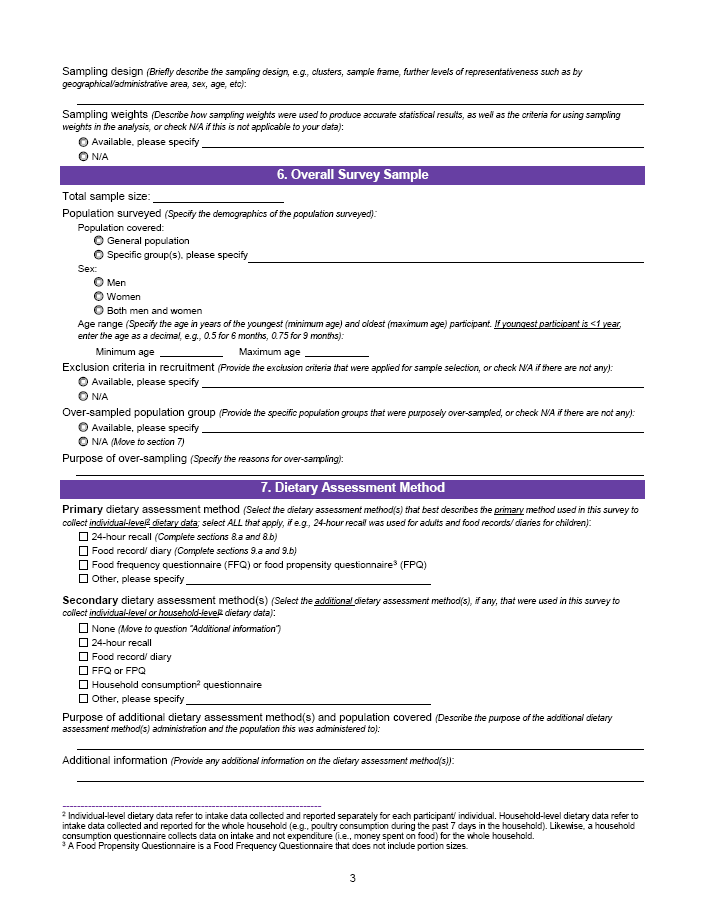


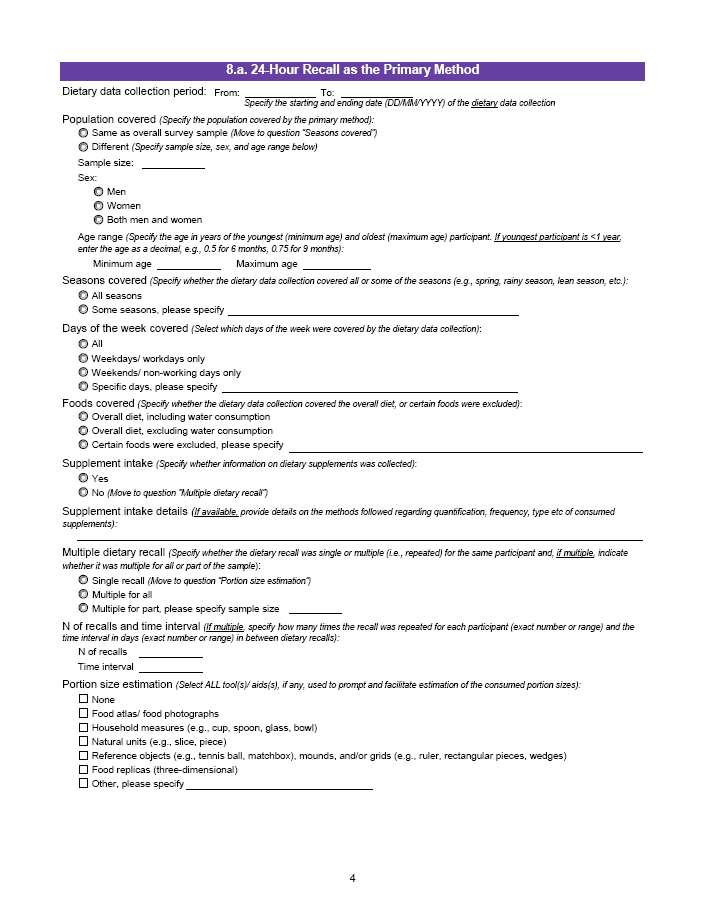


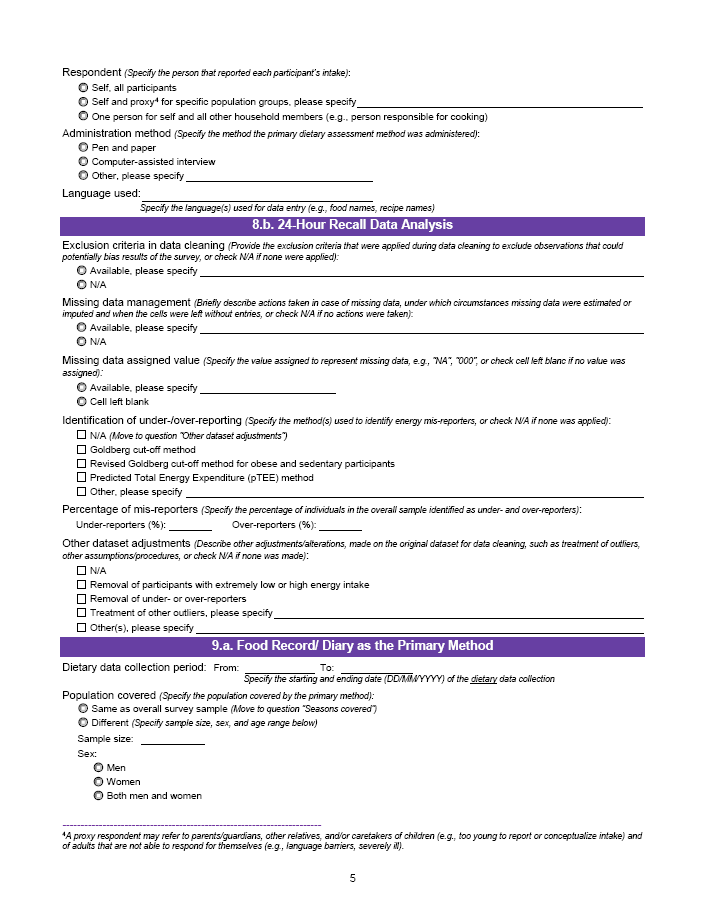


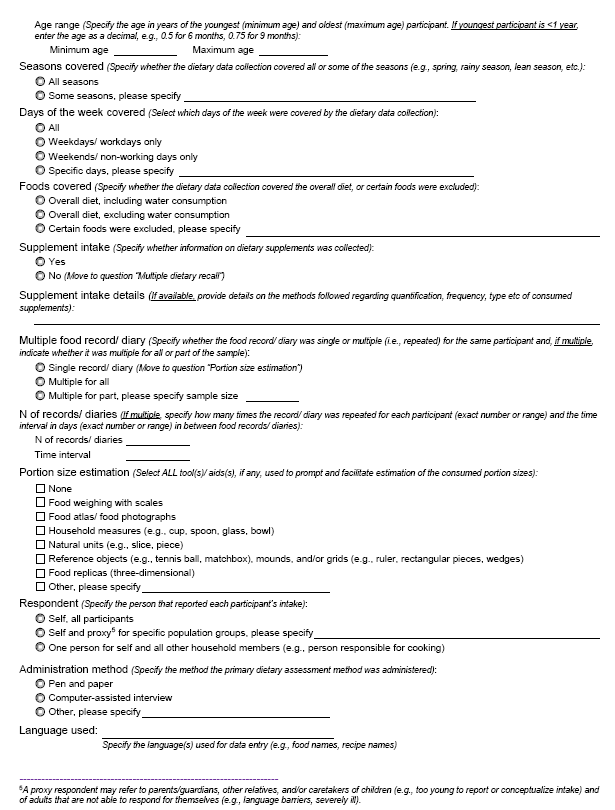


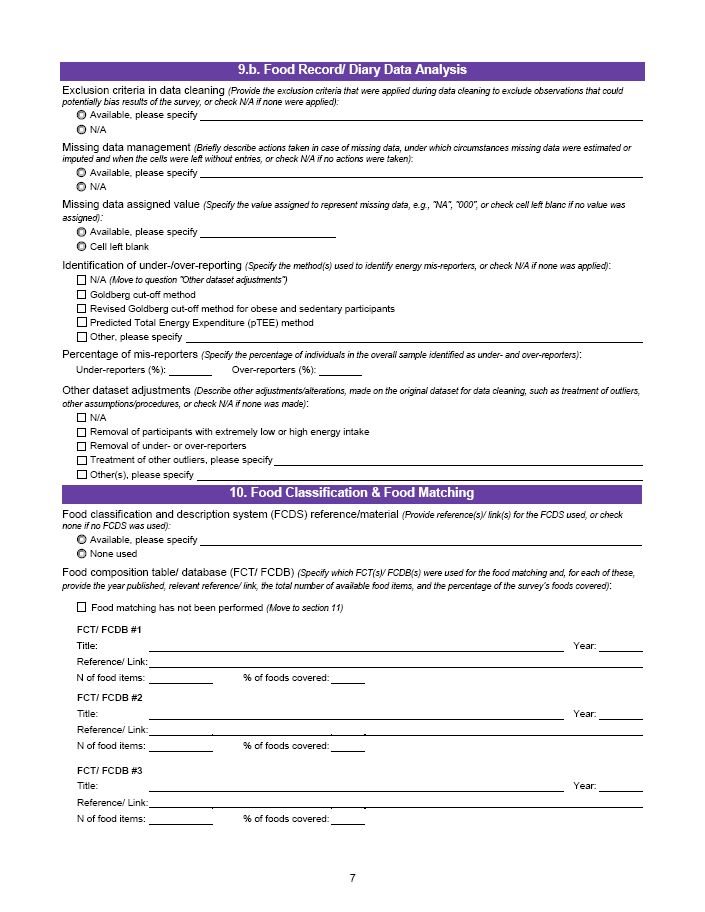


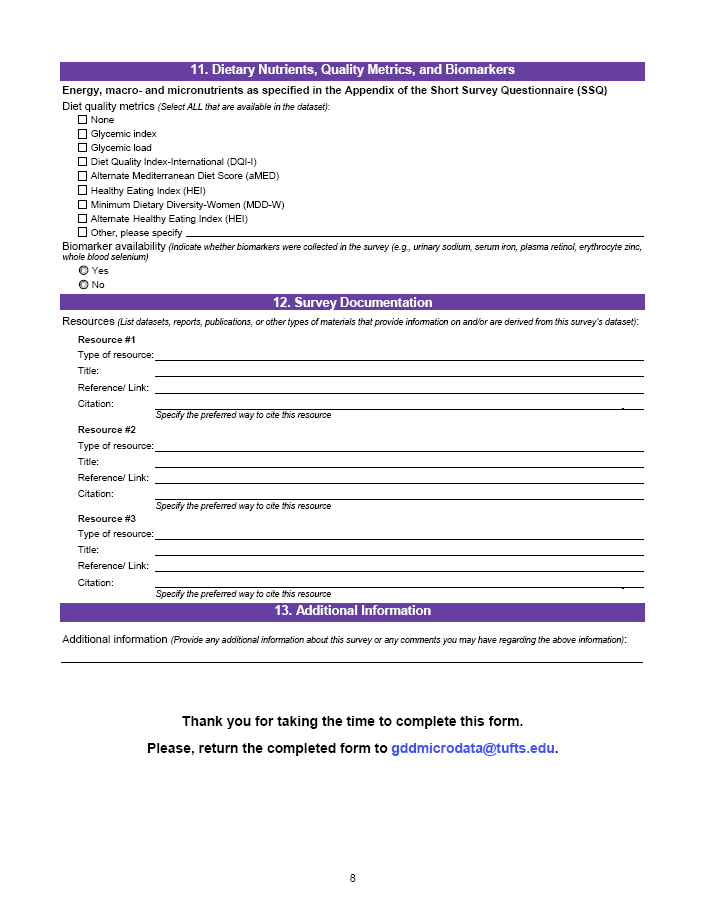


# **File S3.** List of abbreviations.

EFSA, European Food Safety Authority

FAO, Food and Agriculture Organization of the United Nations

FCT, Food composition table

FCDB, Food Composition Database

FCDS, food classification and description system

GDD, Global Dietary Database

GIFT, Global Individual Food consumption data Tool

HIC, High-income countries

LIC, Low-income countries

LMIC, Lower-middle income countries

SSQ, Short Survey Questionnaire

UMIC, Upper-middle income countries

WHO, World Health Organization

# **References**

1. World Bank. Country classification by income. 2020. Available from: <https://datahelpdesk.worldbank.org/knowledgebase/articles/906519-world-bank-country-and-lending-groups>. [Accessed August 2020]

2. European Food Safety Authority. The food classification and description system FoodEx 2 (revision 2). EFSA Supporting Publications. 2015;12(5):804E.
